# Granulocyte-Macrophage Colony Stimulatory Factor Enhances the Pro-Inflammatory Response of Interferon-γ-Treated Macrophages to *Pseudomonas aeruginosa* Infection

**DOI:** 10.1371/journal.pone.0117447

**Published:** 2015-02-23

**Authors:** Sonali Singh, Helen Barr, Yi-Chia Liu, Adrian Robins, Stephan Heeb, Paul Williams, Andrew Fogarty, Miguel Cámara, Luisa Martínez-Pomares

**Affiliations:** 1 School of Life Sciences, University of Nottingham, Nottingham, NG7 2RD, United Kingdom; 2 School of Medicine, University of Nottingham, Nottingham, NG7 2RD, United Kingdom; 3 School of Community Health Sciences, University of Nottingham, Nottingham, NG7 2RD, United Kingdom; University of Pittsburgh, UNITED STATES

## Abstract

*Pseudomonas aeruginosa* is an opportunistic pathogen that can cause severe infections at compromised epithelial surfaces, such those found in burns, wounds, and in lungs damaged by mechanical ventilation or recurrent infections, particularly in cystic fibrosis (CF) patients. CF patients have been proposed to have a Th2 and Th17-biased immune response suggesting that the lack of Th1 and/or over exuberant Th17 responses could contribute to the establishment of chronic *P*. *aeruginosa* infection and deterioration of lung function. Accordingly, we have observed that interferon (IFN)-γ production by peripheral blood mononuclear cells from CF patients positively correlated with lung function, particularly in patients chronically infected with *P*. *aeruginosa*. In contrast, IL-17A levels tended to correlate negatively with lung function with this trend becoming significant in patients chronically infected with *P*. *aeruginosa*. These results are in agreement with IFN-γ and IL-17A playing protective and detrimental roles, respectively, in CF. In order to explore the protective effect of IFN-γ in CF, the effect of IFN-γ alone or in combination with granulocyte-macrophage colony-stimulating factor (GM-CSF), on the ability of human macrophages to control *P*. *aeruginosa* growth, resist the cytotoxicity induced by this bacterium or promote inflammation was investigated. Treatment of macrophages with IFN-γ, in the presence and absence of GM-CSF, failed to alter bacterial growth or macrophage survival upon *P*. *aeruginosa* infection, but changed the inflammatory potential of macrophages. IFN-γ caused up-regulation of monocyte chemoattractant protein-1 (MCP-1) and TNF-α and down-regulation of IL-10 expression by infected macrophages. GM-CSF in combination with IFN-γ promoted IL-6 production and further reduction of IL-10 synthesis. Comparison of TNF-α vs. IL-10 and IL-6 vs. IL-10 ratios revealed the following hierarchy in regard to the pro-inflammatory potential of human macrophages infected with *P*. *aeruginosa*: untreated < treated with GM-CSF < treated with IFN-γ < treated with GM-CSF and IFN-γ.

## Introduction

*Pseudomonas aeruginosa* is an opportunistic, Gram-negative bacterial pathogen that poses a serious healthcare challenge. It can colonise and adapt to many different environments due to its versatile metabolism and ability to form biofilms and produce a wide range of virulence factors [1,2]. Cystic fibrosis (CF) patients are especially susceptible to chronic, often fatal, respiratory infections caused by *P*. *aeruginosa* [3] with this bacterium being considered the primary cause of morbidity and mortality in the CF population [4].

In CF patients, high systemic and pulmonary expression of the T helper cell 2 (Th2) cytokine interleukin 4 (IL-4) correlates with chronic *P*. *aeruginosa* infection and poor pulmonary function, while high expression of the Th1 cytokine interferon-γ (IFN-γ) correlates with improved pulmonary function [5–7]. Infections using animal models also show that IFN-γ or a Th1 bias is protective in *P*. *aeruginosa* respiratory infections [8,9]. It is widely accepted that macrophages present during Th1 and Th2 responses differ greatly: IFN-γ induces M1/classically activated macrophages, which are highly phagocytic, microbicidal against intracellular pathogens, and pro-inflammatory, while the Th2 cytokines IL-4 and IL-13 induce M2/alternatively activated macrophages, which are much less microbicidal and inflammatory [10,11]. CF patients infected with *P*. *aeruginosa* have a higher percentage of M2 macrophages in their lungs than those who are not and this inversely correlates with lung function [12]. In line with this, CF patients infected with *P*. *aeruginosa* who are on azithromycin, a drug shown to induce M2 macrophage activation, present a very high percentage of M2 macrophages and very poor lung function [12] [13].

Recently, CF patients have also been shown to display a Th17-biased immune response that could contribute to deterioration of lung function [14–17] with one report highlighting the co-existence of Th17 and Th2 cytokine profiles in CF [18]. IL-17A is important for protection against extracellular bacterial and fungal pathogens, particularly in mucosal tissue [19]. In particular, IL-17A promotes the generation, recruitment, and activation of neutrophils by inducing the expression of: (i) the colony-stimulating factors: granulocyte colony-stimulating factor (G-CSF) and granulocyte-macrophage colony-stimulating factor (GM-CSF), and (ii) the chemokines: IL-8, growth-related oncogene α (GRO-α), and granulocyte chemotactic protein 2 (GCP-2) [20]. It has been suggested that overproduction of IL-17A may drive chronic pulmonary inflammation in CF [21].

Macrophages play important roles both as immunomodulatory cells that influence inflammatory responses and as effector cells that phagocytose and kill pathogens [22]. Numerous studies have demonstrated that human and murine macrophages phagocytose *P*. *aeruginosa* [23–25] and that murine macrophages produce cytokines and chemokines in response to *P*. *aeruginosa* or its products [26,27]. An important consideration is how macrophage activation affects responses to *P*. *aeruginosa*.

The study presented in this manuscript shows that IFN-γ production by peripheral blood mononuclear cells (PBMCs) from CF patients positively correlates with lung function, whereas the opposite is the case for IL-17A production. We hypothesised that IFN-γ could be protective in CF by modifying the response of macrophages to *P*. *aeruginosa* infection which in turn could affect the inflammatory response in the infected lung. To test this, the effect of IFN-γ on the interaction of *P*. *aeruginosa* with primary human macrophages from healthy donors was investigated. GM-CSF was also used to treat macrophages because: (i) it has an activating effect on myeloid cell function [28,29]; (ii) it has therapeutic potential in CF [30]; (iii) high serum GM-CSF levels correlate with increased IFN-γ expression and better pulmonary function in CF patients [6], and (iv) GM-CSF complements IFN-γ in macrophage resistance to mycobacterial infection [31], strongly suggesting that the two factors may co-operate in host defence.

Our results show that IFN-γ in the presence and absence of GM-CSF does not alter the ability of human macrophages to control *P*. *aeruginosa* growth or improve macrophage survival during infection. IFN-γ promotes the pro-inflammatory response of human macrophages to *P*. *aeruginosa* infection by increasing the TNF-α vs IL-10 and IL-6 vs IL-10 ratios and these effects are enhanced by GM-CSF. This study provides further information on what constitutes a beneficial immune response against *P*. *aeruginosa* in humans and how cytokine(s)-based immunotherapy [32] could influence effector immune responses against this pathogen.

## Materials and Methods

### Ethics statement

Study using two groups of CF patients and control PBMCs was approved by the Nottingham University Hospital NHS Trust Research and Development (ID: 09RM001) and the University of Nottingham Medical School Research Ethics Committee (Ethics Reference No: BT28092010), respectively. All donors provided informed written consent and samples were anonymised.

### CF patients and controls

Study subjects were ≥ 18 years old. In the present study, CF patients were divided in two groups to compare those with chronic P. aeruginosa infection (referred to in this study as CF/Chronic PA) versus those with intermittent infection or free of infection (referred to in this study as CF/Intermittent-Free PA). P. aeruginosa infection status was defined according to the Leeds criteria [33]. CF patients were clinically stable and not on intravenous antibiotics at the time of the study. Healthy volunteers were age-, sex-, and ethnicity-matched to patients had no history of respiratory disease, were not suffering from infection or on medication at the time of sample collection. See Table 1 for demographic details for CF patients, including classification criteria, and matched healthy controls utilised in the present study.

**Table 1:** Demographic details for CF patients and matched healthy controls utilised in the present study.

	Healthy controls	CF/Chronic PA	CF/Intermittent-Free PA
**Number (n)**	13	15	15
			Intermittent n = 2
			Free n = 11
			Never n = 2
**Classification criteria [33]**	N/A	Chronic: > or = 50% of cultures positive for *P*. *aeruginosa* in past 12 months	Intermittent: <50% cultures positive for *P*. *aeruginosa* in past 12 months
			Free: *P*. *aeruginosa* not isolated in past 12 months but has been isolated prior to this study
			Never: *P*. *aeruginosa* never isolated
**Sex (F/M)**	6/7	6/9	8/7
**Mean age ± SD (years)**	29.6 ± 4.8	27.7 ± 8.1	27.5 ± 11.4
**Genotype**	N/A	ΔF508 homozygous: 6	ΔF508 homozygous: 7
		ΔF508 heterozygous: 4	ΔF508 heterozygous: 2
		Other: 2	Other: 1
		Unknown: 3	Unknown: 5

F, female; M, male; SD, standard deviation; N/A, not applicable.

### Assessment of lung function

Spirometry was performed using a micro-medical microlab (ML 3500) spirometer according to the joint ERS/ATS criteria [34].

### Preparation of PAO1-L lysates for PBMC stimulation

The strain of *P*. *aeruginosa* PAO1, subline Lausanne (PAO1-L), was used throughout this study. PAO1 is a serogroup O2/O5, type b flagellated strain [33,34] exhibiting moderate virulence [35]. The genome of PAO1-L has been fully sequenced and found closest to Holloway’s original isolate (data not shown) [36,37]. Mid-log phase cultures of PAO1-L (3 hour, h) in Luria-Bertani (LB) broth were washed with PBS without Ca^2+^ or Mg^2+^ (Sigma-Aldrich), resuspended in RPMI-1640 without phenol red (Sigma-Aldrich) such that cultures were 20x concentrated, and lysed using a French Press. Lysates were centrifuged to remove debris, filtered through a 0.2 μm filter, aliquoted, snap frozen on dry ice, and stored at -80°C until used. Total protein content in lysates was quantified using the bicinchoninic acid assay (Thermo Scientific).

### PBMC stimulation and culture

Venous blood was collected in EDTA vacutainers (BD Diagnostics-Preanalytical Systems), the plasma was removed, and cells were processed within 24 h. 2 x 10^5^ PBMCs in 100 μl/well were cultured in RPMI-1640 containing 10% human AB serum, 2 mM L-glutamine, 100 U/ml penicillin, 100 μg/ml streptomycin, and 10 mM HEPES in 96-well U-bottom tissue culture plates (Corning Life Sciences) and stimulated with 2% (v/v) phytohaemagglutinin (PHA, M-form, Invitrogen), 10 ng/ml staphylococcal enterotoxin B (SEB, Sigma-Aldrich) or two concentrations (6,250 ng/ml and 24 ng/ml total protein) of two independently prepared PAO1-L lysates at 37°C, 5% CO_2_ for 6 days. On Day 6 PBMCs were re-stimulated with 15 ng/ml phorbol 12-myristate 13-acetate (PMA, Sigma-Aldrich) overnight, and supernatants harvested for cytokine analysis on Day 7.

### Generation and activation of monocyte-derived macrophages

PBMCs were isolated from buffy coats (Blood Transfusion Service, Sheffield, UK) by Histopaque-1077 (Sigma-Aldrich) density gradient centrifugation, and monocytes purified using human CD14 MicroBeads and LS MACS columns (Miltenyi Biotec). Monocytes were cultured in Teflon bottles (Nalgene, Fisher Scientific, 10^7^ monocytes/ bottle) in RPMI-1640 (Sigma-Aldrich) containing 15% human AB serum (Sigma-Aldrich or PAA Laboratories Ltd), 2 mM L-glutamine, 100 U/ml penicillin, 100 μg/ml streptomycin (Sigma-Aldrich), 10 mM HEPES (Invitrogen), and 50 ng/ml human macrophage colony-stimulating factor (M-CSF, R&D Systems) at 37°C, 5% CO_2_ for 7 days. Fresh medium containing 50 ng/ml M-CSF was added on Day 3. Macrophages expressed the expected surface markers (S1A and B Fig.) and generated oxidative burst products in response to zymosan (S1C Fig.). On Day 7, macrophages were plated on 24-well tissue culture plates (Corning Life Sciences) at 2.5 x 10^5^ macrophages per well in X-Vivo 15 (Lonza) with M-CSF (50 ng/ml) or GM-CSF (50 ng/ml, Miltenyi Biotec), in the presence and absence of IFN-γ (200 U/ml = 10 ng/ml, R&D Systems) and cultured at 37°C, 5% CO_2_ for 48 h.

### Inocula preparation

To avoid potential interference from microbe-associated molecular patterns (MAMPs) and danger-associated molecular patterns (DAMPs) [35] contained in commonly used bacterial growth media such as LB broth, *P*. *aeruginosa* inocula were grown and prepared in the same serum-free medium (X-Vivo 15) in which the infections were performed. *P*. *aeruginosa* growth and expression of key quorum sensing regulatory genes and quorum sensing controlled genes encoding virulence factors in X-Vivo 15 were evaluated and found suitable for the study (S2 and S3 Figs. and S1 Table). *P*. *aeruginosa* strain PAO1-L from glycerol stocks was streaked on to an LB agar plate and incubated at 37°C overnight. The following day a single colony was inoculated into 5 ml of X-Vivo 15 and incubated overnight at 37°C, 200 rpm. The overnight culture was adjusted to OD_600nm_ = 1, diluted 1:100 in 25 ml of fresh X-Vivo 15 in a 250 ml flask, and incubated at 37°C, 200 rpm for 3 h. This 3 h culture was washed twice with cold PBS without Ca^2+^ or Mg^2+^, resuspended in X-Vivo 15, counted using a Thoma bacterial counting chamber (Hawksley) and a Nikon Eclipse TE200 microscope, and the density of the culture adjusted to achieve a multiplicity of infection (MOI) = 1.

### Macrophage infection

Macrophages plated in 24-well plates were washed three times with cold PBS without Ca^2+^ or Mg^2+^. PAO1-L mid-log phase inoculum containing the appropriate cytokine(s) was then added to the wells (MOI = 1, 350 μl inoculum per well) and cultures were incubated at 37°C, 5% CO_2_ for up to 6 h. Since *P*. *aeruginosa* is mainly an extracellular pathogen, non-internalised bacteria were maintained throughout the course of the infection. Controls were uninfected macrophages treated with the same cytokines, and bacteria only cultures—wells that contained the PAO1-L inoculum with the appropriate cytokine(s) but no macrophages. To measure the macrophage-associated bacteria, macrophages were lysed by adding cold ultra-pure distilled water (Invitrogen). Appropriate dilutions of the macrophage supernatants and lysates and bacteria only controls were prepared in PBS and plated on LB agar plates and incubated at 37°C overnight to determine viable cfu. Both extracellular and macrophage-associated bacteria were expressed as a percentage of the bacteria only controls for that time point.

### Quantification of macrophage cytotoxicity

Lactate dehydrogenase (LDH) activity was measured using the Cytotoxicity Detection Kit (Roche Applied Science) as per the manufacturer’s instructions. A standard curve prepared from an LDH positive control (Promega) was used to calculate the LDH concentration in samples. Lysates of macrophages prepared using 0.1% Triton X-100 were used as 100% lysis controls. Bacterial contribution to LDH readings was negligible (S4 Fig.)

### Microscopic assessment of infected macrophages

Day 7 macrophages plated on sterile, acid-etched coverslips were activated and infected as described above. At the required time points post-infection supernatants were removed and cells fixed in 4% paraformaldehyde (Electron Microscopy Science) in PBS. Fixed samples were stained using the Hemacolor Staining Kit (Merck Millipore) as per the manufacturer’s instructions, mounted on slides using DPX mounting medium (Raymond A Lamb Laboratory Supplies), viewed using a Zeiss Axioplan light microscope, and images captured with a Q Imaging MicroPublisher 5.0 RTV camera and Openlab software.

### Cytokine quantification

Cytokines and chemokines in supernatants from PBMC stimulation assays and macrophages infection assays were quantified using the FlowCytomix bead-based assay (eBioscience). Supernatants had to be diluted 1:10 in order to measure TNF-α and IL-18 accurately as these cytokines were produced at quantities that exceeded the standard range of the respective cytokine kits. Results were analysed using the eBioscience FlowCytomix Pro 2.4 software.

### Statistical analysis

Statistical analyses were performed in GraphPad Prism v 6.02. For CF and healthy control PBMC cytokine responses significance was calculated using Kruskal-Wallis test with Dunn’s post test. Correlations between CF pulmonary function and cytokine responses were calculated by Spearman rank test. For macrophage experiments significance was calculated by one-way ANOVA with Tukey’s post test (for unpaired data) or repeated measures one-way ANOVA with Tukey’s or Dunnett’s post test (for paired data). LDH release at different times post-infection was analysed using a paired Student’s t test. p≤0.05 was considered significant in all cases.

## Results

### IFN-γ and IL-17A production by stimulated PBMC cultures from CF patients and healthy controls

**Fig 1:**
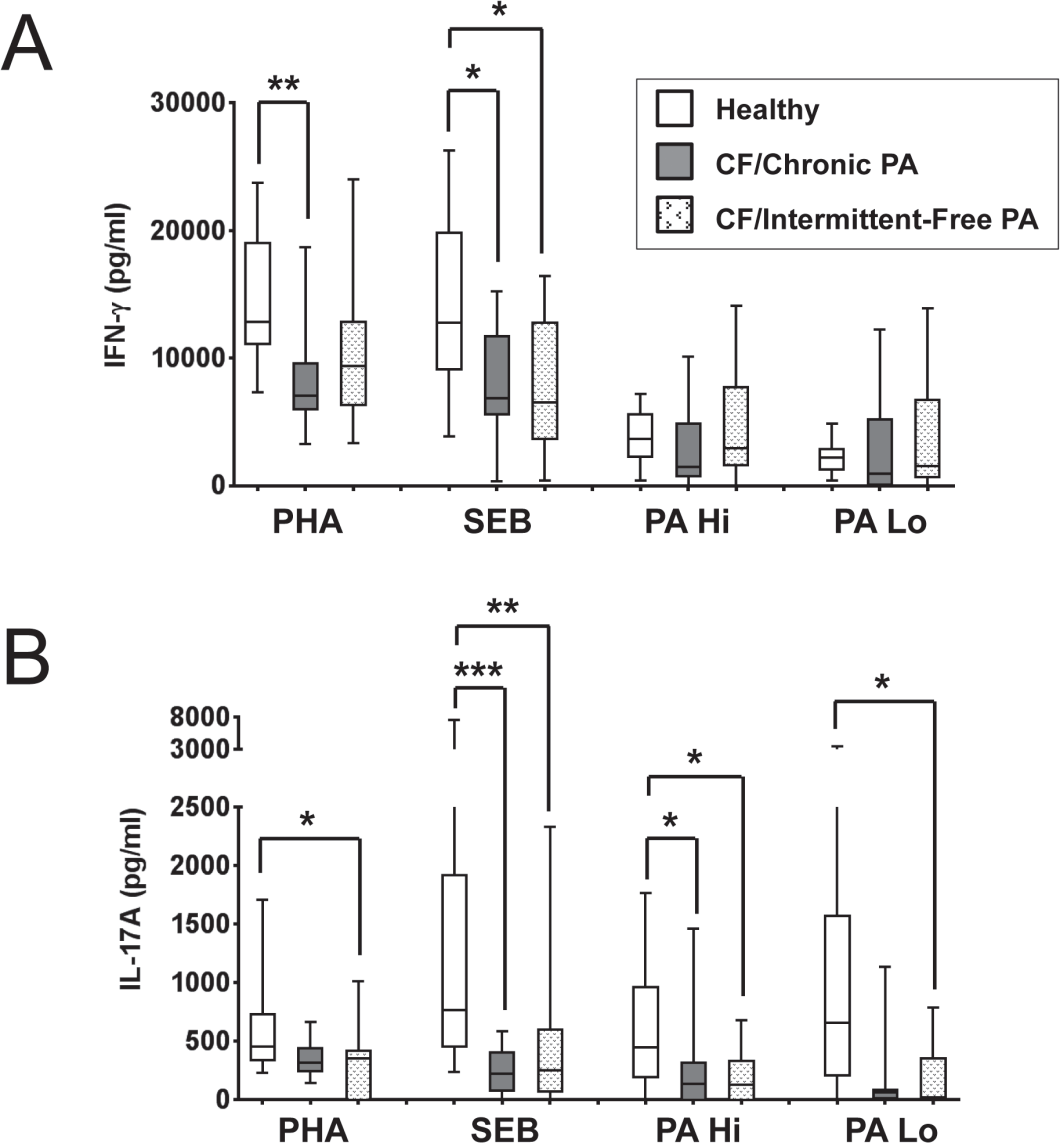
Production of IFN-γ and IL-17A in CF and control PBMCs in response to several stimuli. PBMCs were stimulated with 2% (v/v) phytohaemagglutinin (PHA), 10 ng/ml staphylococcal enterotoxin B (SEB), or two concentrations of PAO1-L lysates—PA Hi (6,250 ng/ml total protein) and PA Lo (24 ng/ml total protein)—for 6 days, re-stimulated with 15 ng/ml phorbol 12-myristate 13-acetate (PMA) overnight, and supernatants collected on day 7 for cytokine analysis. **A**. CF patient PBMCs produced less IFN-γ than healthy control PBMCs in response to PHA and SEB, but not PAO1-L lysates. **B**. CF patient PBMCs produced less IL-17A than healthy control PBMCs in response to PHA, SEB and PAO1-L lysates. Graph depicts 5–95 percentile with median; healthy controls: n = 13, CF/Chronic PA and CF/Intermittent-Free PA: n = 15 each. Significance calculated by Kruskal-Wallis test with Dunn’s post test; * = p≤0.05, ** = p≤0.01, *** = p≤0.001.

Previous studies have demonstrated attenuated IFN-γ or Th1 responses and enhanced IL-17A or Th17 responses in CF patient peripheral blood and lungs [5–7,14–18], however they did not establish whether there were any differences in these responses between patients chronically or intermittently infected with *P*. *aeruginosa*. Therefore, in this study the production of IFN-γ (Fig. 1A), IL-17A (Fig. 1B), IL-13 and IL-10 (S5 Fig.) in an independent CF population consisting of patients with either chronic or intermittent/free *P*. *aeruginosa* infections (CF/Chronic PA and CF/Intermittent-Free PA, respectively) was investigated. PBMCs from CF patients and healthy controls were stimulated as described in materials and methods with the mitogen phytohaemagglutinin, PHA, the superantigen staphylococcal enterotoxin B,SEB, and two doses of *P*. *aeruginosa* (PAO1-L) lysates (PA Hi and PA Lo) (See Table 1 for demographic details for patients and healthy volunteers). *P*. *aeruginosa* lysates were selected as stimuli because CF patients were stratified based on chronicity of *P*. *aeruginosa* infection in this study and similar preparations have previously been used by other authors [18]. The overall IFN-γ production was reduced by approximately 1.5 to 2-fold in both groups of CF patients as compared to healthy controls when PBMCs were stimulated with PHA or SEB (Fig. 1A). For CF/Chronic PA patients, this attenuation was significant in response to both PHA (p = 0.0014) and SEB (p = 0.0356), while for CF/Intermittent-Free PA patients, it was only significant in response to SEB (p = 0.0386). Levels of IL-17A were also consistently reduced in CF PBMC cultures compared to healthy controls (Fig. 1B). The IL-17A reduction was significant for CF/Chronic PA patients in response to SEB (p = 0.0007) and PA Hi (p = 0.0386), and for CF/Intermittent-Free PA patients in response to PHA (p = 0.0420), SEB (p = 0.0096), PA Hi (p = 0.0292) and PA Lo (p = 0.0137). The production of IL-13 and IL-10 was not consistently reduced in the CF samples (S5 Fig.) which argues against the attenuation in IFN-γ and IL-17A production being due to greater cell death in these PBMC cultures.

### Correlation between IFN-γ and IL-17A production by stimulated PBMC cultures and lung function in CF patients

**Fig 2:**
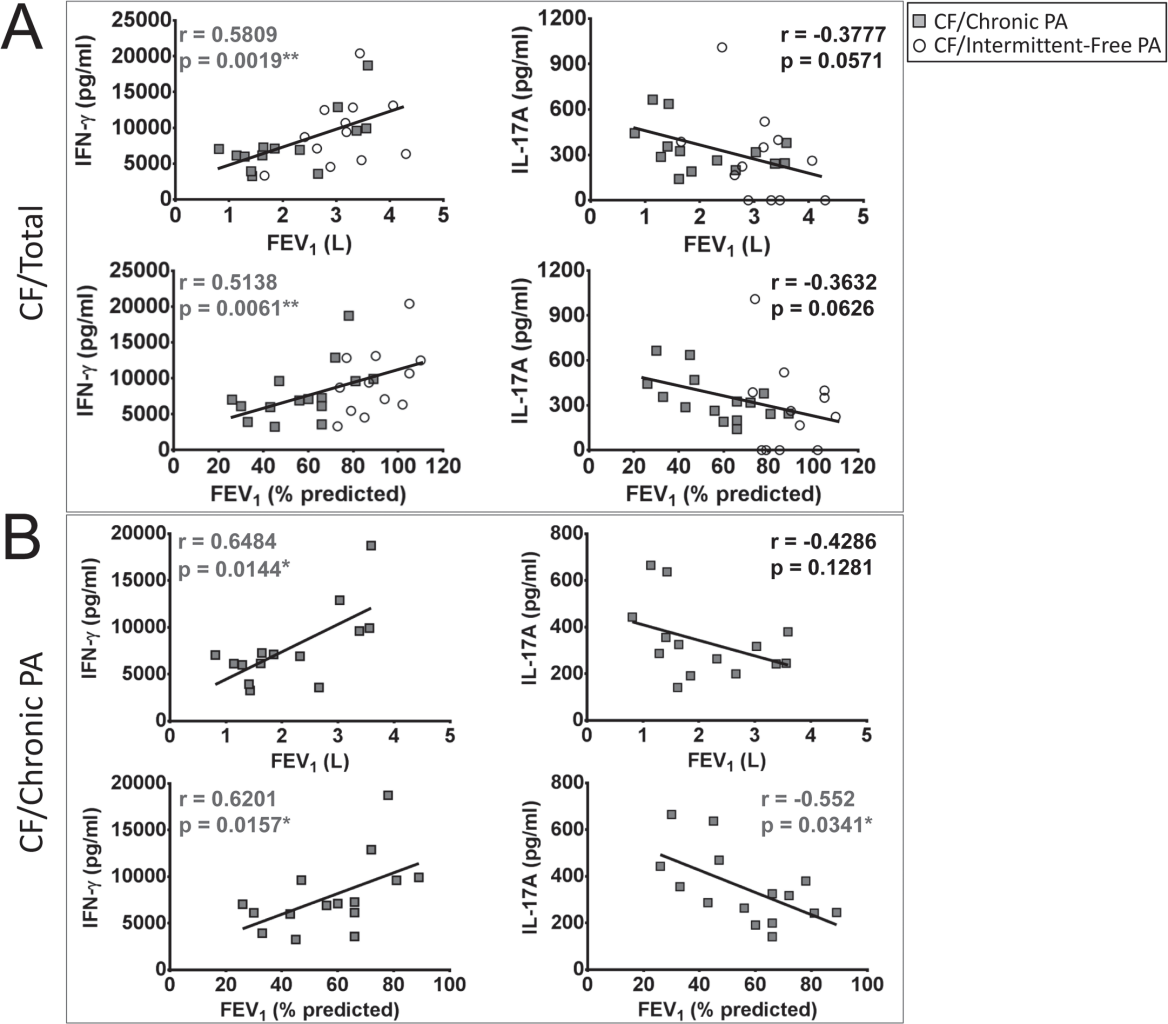
Correlation between IFN-γ and IL-17A production by PHA-stimulated PBMC cultures and lung function in CF patients. **A**. Highly significant positive correlation was observed between IFN-γ production in response to phytohaemagglutinin (PHA) and lung function in CF patients as measured by absolute and % predicted forced expiratory volume in one second (FEV_1_). A trend towards a negative correlation was observed between IL-17A production in response to PHA and lung function in CF patients as measured by absolute and % predicted FEV_1_. **B**. Significant positive correlation between IFN-γ expression by CF/Chronic PA PBMCs in response to PHA with lung function as measured by absolute and % predicted FEV_1_. Significant negative correlation between IL-17A expression by CF/Chronic PA PBMCs in response to PHA with lung function as measured by % predicted FEV_1_. CF/Chronic PA: n = 14 (as lung function data was not available for 1 of the 15 CF/Chronic PA patients), CF/Intermittent-Free PA: n = 12 (as lung function data was not available for 3 of the 15 CF/Intermittent-Free PA patients). Spearman test was used to determine correlation; * = p≤0.05, ** = p≤0.01.

To ascertain the potential clinical relevance of the levels of IFN-γ and IL-17A produced by stimulated CF PBMCs, their possible correlation with pulmonary function in these patients was studied. When the CF patients were considered as a single population, a highly significant positive correlation was observed between pulmonary function (measured as forced expiratory volume in one second, FEV_1_) and IFN-γ production by PBMCs in response to PHA (Fig. 2A) (IFN-γ vs. FEV_1_ (L): r = 0.5809, p = 0.0019; IFN-γ vs. % predicted FEV_1_: r = 0.5138, p = 0.0061). CF/Intermittent-Free PA patients had significantly better lung function than CF/Chronic PA patients (S6 Fig.), and when both groups of CF patients were analysed separately, the correlation between IFN-γ production in response to PHA and lung function only remained significant in the case of CF/Chronic PA patients (Fig. 2B; S7 Fig.) (CF/Chronic PA patients, IFN-γ vs. FEV_1_ (L): r = 0.6484, p = 0.0144; IFN-γ vs. % predicted FEV_1_: r = 0.6201, p = 0.0157). A positive, significant correlation between levels of IFN-γ produced by CF/Chronic PA PBMCs in response to PA Hi and FEV_1_ (absolute, r = 0.5611, p = 0.0394 and % predicted, r = 0.5256, p = 0.0465) was also observed. Levels of IFN-γ in response to PA Lo did not correlate with lung function (data not shown).

In the case of IL-17A, a trend towards a negative correlation between IL-17A levels in response to PHA and lung function was observed when the CF patients were considered as a single population (Fig. 2A), and this trend became significant only when CF/Chronic PA patients were considered and FEV_1_ was expressed as % predicted (Fig. 2B; S8 Fig.) (CF/Chronic PA patients, IFN-γ vs. % predicted FEV_1_: r = -0.552, p = 0.0341). IFN-γ and IL-17A production in response SEB and IL-17A production in response to PA lysates did not correlate with lung function (data not shown).

These results are consistent with an association between chronic *P*. *aeruginosa* infection and poor lung function in CF patients, and with a protective role for IFN-γ and a detrimental role for IL-17A in CF, particularly when lung function declines.

### Pro-inflammatory response of human macrophages to *P*. *aeruginosa* infection

The protective role of IFN-γ in CF led to investigate how this cytokine could assist in the maintenance of lung function in the context of *P*. *aeruginosa* infection. Given that macrophage activation appears to correlate with *P*. *aeruginosa* infection status and lung function in CF patients [12], the impact of IFN-γ treatment on the interaction of healthy human macrophages with *P*. *aeruginosa* was investigated. Towards this aim, the response of untreated macrophages to *P*. *aeruginosa* infection was studied in the first instance. Human macrophages were infected at MOI = 1 as described in materials and methods and the following parameters were analysed at 2, 4, and 6 hours post-infection (hpi): cytokine production by macrophages, bacterial cfu both in the supernatants and cell-associated fraction, and macrophage death.

**Fig 3:**
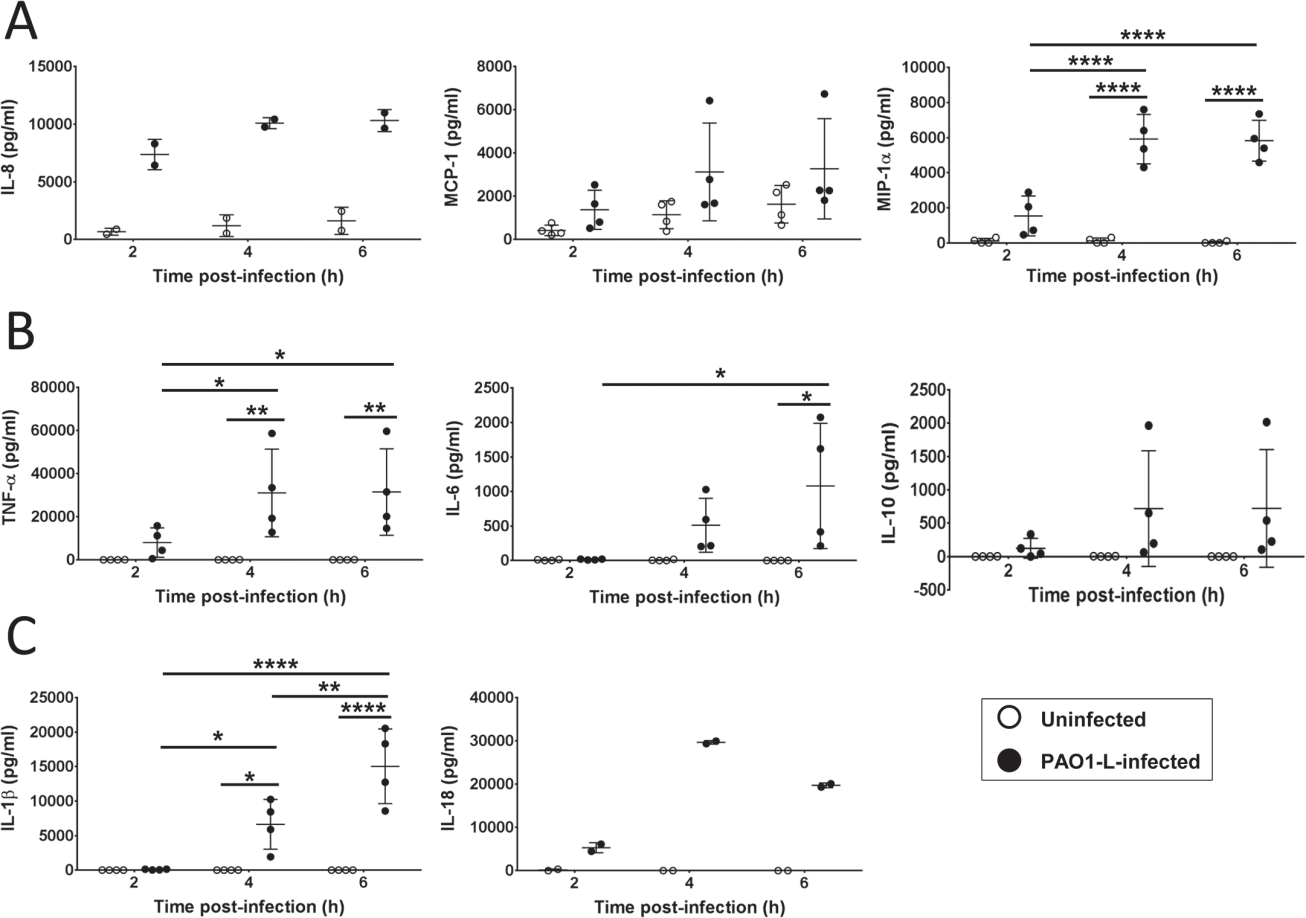
Production of chemokines and cytokines by human macrophages upon infection with *P*. *aeruginosa*. Supernatants from uninfected and infected macrophages (MOI = 1) were collected at 2, 4, and 6 hpi and tested for the presence of IL-8, MCP-1, and MIP-1α **(A)** TNF-α, IL-6 and IL-10 **(B)** and IL-1β and IL-18 **(C)**. IL-8 and MCP-1 were detected in supernatants from uninfected macrophages. All cytokines and chemokines tested were upregulated upon PAO1-L infection, with most peaking at 4 hpi. Data presented are mean ± SD for n = 4 for all cytokines except IL-8 and IL-18 where n = 2. Significance calculated by repeated measures one-way ANOVA with Tukey’s post test; * = p≤0.05, ** = p≤0.01, **** = p≤0.0001.

Several cytokines and chemokines were quantified in supernatants to obtain a general overview of the type of inflammatory response elicited by live *P*. *aeruginosa* in human macrophages. These include chemoattractants for neutrophils (IL-8, also termed CXCL8) and monocytes (MCP-1 and MIP-1α, also termed CCL2 and CCL3, respectively) and pro-inflammatory (TNF-α, IL-6, IL1-β and IL-18) and anti-inflammatory (IL-10) cytokines (Fig. 3). IL-8 and MCP-1 were found to be constitutively expressed by uninfected macrophages and their levels increased upon *P*. *aeruginosa* infection, at which time MIP-1α production was also detected (Fig. 3A). PAO1-L infection stimulated a rapid and vigorous cytokine response in macrophages (Fig. 3B). In addition to TNF-α and IL-6, *P*. *aeruginosa* infection also induced IL-10 expression but it was generally low except in one donor (Fig. 3B). Inflammasome activation was indicated by the production of both IL-1β and IL-18 (Fig. 3C). Expression of IL-8, MCP-1, MIP-1α TNF-α, IL-10 and IL-18, peaked at 4 hpi, while that of IL-6 and IL-1β expression continued to increase up to 6 hpi, which was the last time-point measured in this study. The experiments were not continued beyond 6 hpi as widespread damage of the macrophage monolayer was observed microscopically (see below).

**Fig 4:**
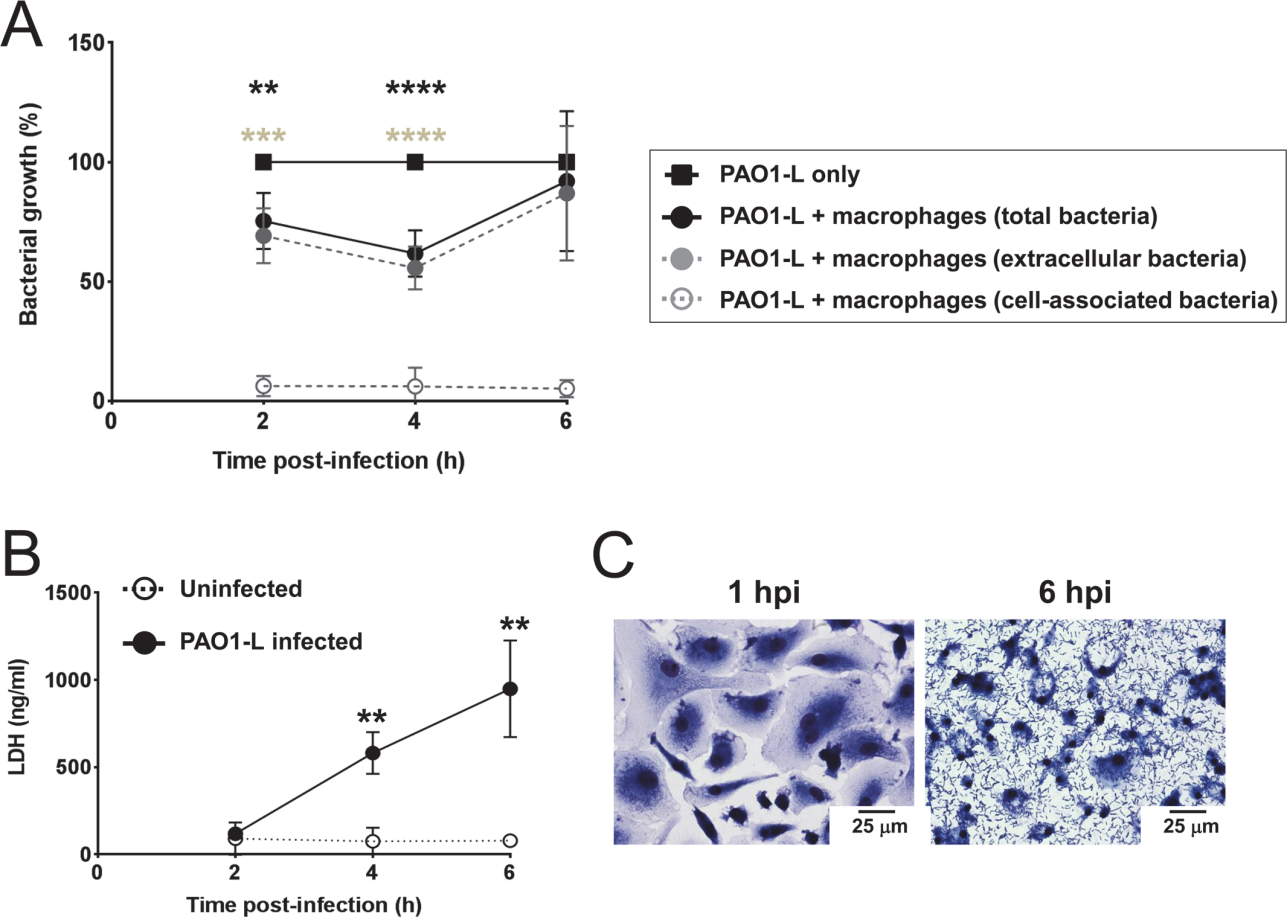
Human macrophages limit PAO1-L growth transiently but undergo significant cell death. **A**. Bacterial growth in the presence and absence of macrophages was quantified at 2, 4, and 6 hpi. In the presence of macrophages, a significant reduction in total (significance reported as black asterisks) and extracellular (significance reported as grey asterisks) PAO1-L was observed at 2 hpi (extracellular bacteria: p = 0.0005; total bacteria: p = 0.0022) and 4 hpi (extracellular and total bacteria: p<0.0001), with maximum reduction at 4 hpi. However, this inhibition was transient and by 6 hpi there were no significant differences between extracellular or total PAO1-L growth in the presence or absence of macrophages (extracellular bacteria: p = 0.5618; total bacteria: p = 0.8323). At all three time points bacteria in infected macrophage wells were predominantly planktonic rather than cell-associated. Data presented are mean ± SD for n = 4. Significance was calculated by repeated measures one-way ANOVA with Dunnett’s post test; ** = p≤0.01, *** = p≤0.001, **** = p≤0.0001. **B**. LDH release at 4 and 6 hpi by infected macrophages was significantly higher than by uninfected macrophages (4 hpi: p = 0.0072; 6 hpi: p = 0.0067). Data presented are mean ± SD for n = 4. Significance calculated by a paired Student’s t test; ** = p≤0.01. **C**. Analysis of infected macrophage cultures by light microscopy showed a monolayer of healthy, phenotypically heterogeneous macrophages with very few bacteria visible at 1 hpi. By 6 hpi, however, *P*. *aeruginosa* dominated the culture and macrophages showed a reduction in the size of nuclei and disappearance of cytoplasm. Magnification = 400x.

Bacterial growth during infection was assessed to determine if macrophages could impact on bacterial replication. Reduced viable bacteria were consistently observed at 2 to 4 hpi (Fig. 4A). At 6 hpi a tendency towards increased bacterial growth was observed and, in two donors, the total bacteria in the presence of macrophages equalled or exceeded the bacteria in control wells. Most bacteria were planktonic with only a small proportion detected in the cell-associated fraction (Fig. 4A). Phagocytosis by macrophages was minimal probably due to the absence of opsonins and low MOI used. Progressive macrophage death occurred, evidenced by increasing LDH levels in supernatants and microscopic analysis of cells (Fig. 4B and C; S8 Fig.).

Thus, *P*. *aeruginosa* infection induced a robust pro-inflammatory cytokine response in untreated human macrophages. Macrophages temporarily restricted *P*. *aeruginosa* growth but widespread macrophage death occurred in spite of the low MOI used.

### GM-CSF in combination with IFN-γ enhances the pro-inflammatory response of human macrophages to P. aeruginosa

**Fig 5:**
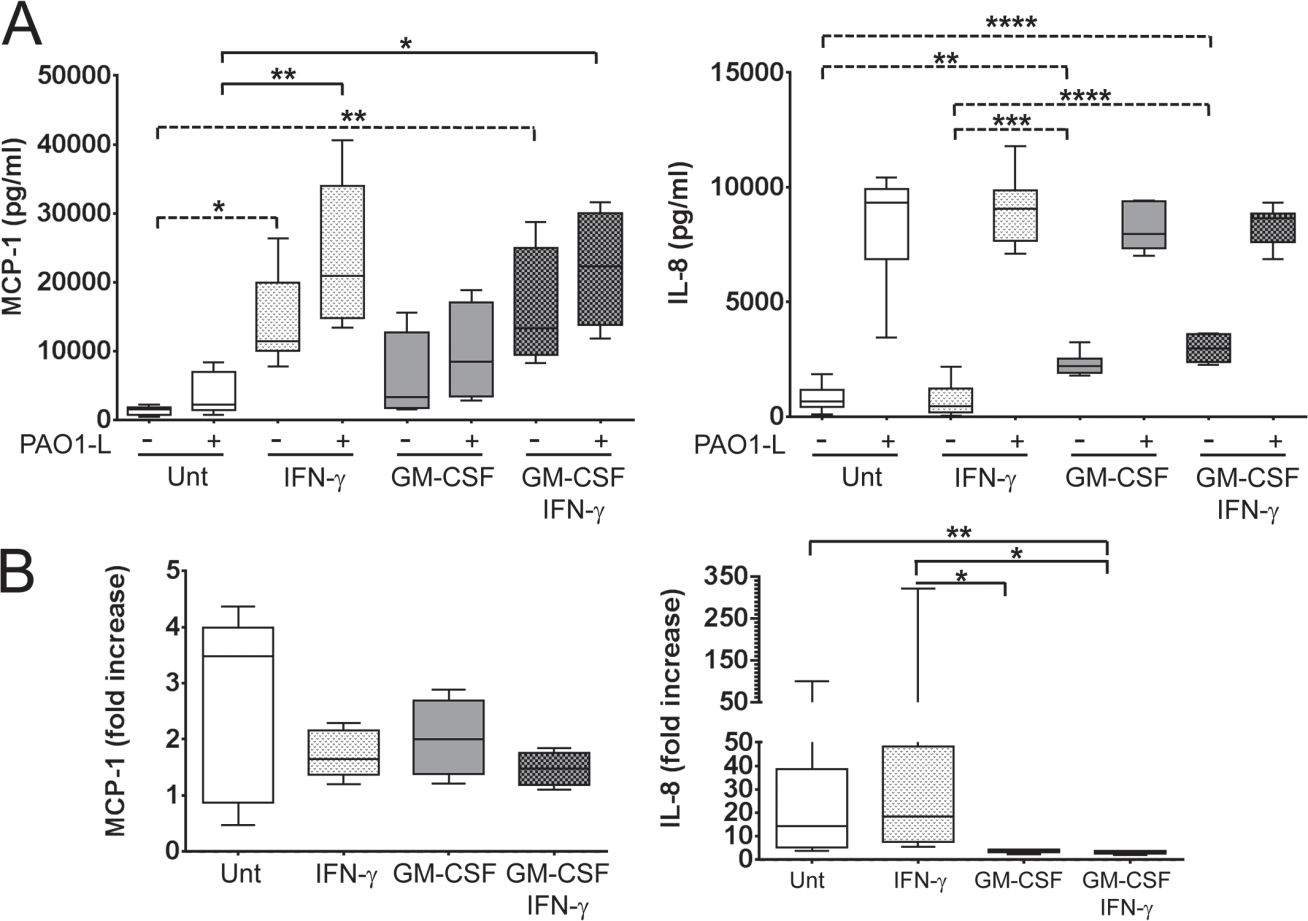
Differential production of MCP-1 and IL-8 by macrophages under steady state and in response to *P*. *aeruginosa* infection upon activation with IFN-γ in the presence and absence of GM-CSF. Macrophages were plated in X-Vivo 15, treated with IFN-γ in the presence and absence of GM-CSF for 48 h and infected with PAO1-L for 4 h. **A**. IFN-γ activation significantly increased expression of MCP-1 in uninfected and PAO1-L-infected macrophages. In contrast, constitutive IL-8 production was up-regulated by GM-CSF but was not significantly altered by macrophage activation after PAO1-L infection. Dashed lines indicate significant differences between responses of uninfected macrophages; solid lines indicate significant differences between responses of PAO1-L-infected macrophages. **B**. Fold increase in MCP-1 and IL-8 expression in infected macrophages compared with uninfected macrophages. Upon infection the median IL-8 expression by untreated and IFN-γ-treated macrophages was up-regulated by 14-fold and 18-fold above baseline levels, respectively, while that by macrophages treated with GM-CSF in the presence and absence of IFN-γ only increased by 3 to 4-fold above baseline levels. No significant differences were observed in MCP-1 up-regulation among the four macrophage populations. Please note the differences in scale between the lower (0–50) and higher values (50–350) in the graph representing IL-8 fold increase. Graphs depict 5–95 percentile with median for n = 4 to 8. Significance calculated by one-way ANOVA with Tukey’s post test. * = p≤0.05, ** = p≤0.01, *** = p≤0.001, **** = p≤0.0001. Unt: untreated macrophages; (-): uninfected; (+): infected.

To further investigate the contribution of human macrophages to the positive correlation between IFN-γ and lung function in CF in the context of *P*. *aeruginosa* infection, the ability of IFN-γ to enhance the response of macrophages to *P*. *aeruginosa* infection was studied. The pro-inflammatory cytokine GM-CSF was used in addition to IFN-γ to treat macrophages. Thus, four types of differentially activated macrophages were investigated in the present study: untreated macrophages, macrophages treated with IFN-γ, macrophages treated with GM-CSF, and macrophages treated with GM-CSF and IFN-γ. The effect of cytokine treatment on the interaction of *P*. *aeruginosa* and human macrophages was studied at 4 hpi based on results obtained with untreated macrophages (Fig. 3). The four types of differentially activated macrophage populations differed in their ability to produce chemokines in the absence of infection (Fig. 5). IFN-γ significantly increased constitutive MCP-1 expression (IFN-γ: p = 0.0106; GM-CSF and IFN-γ: p = 0.0098), while GM-CSF significantly increased constitutive IL-8 expression (GM-CSF: p = 0.0020; GM-CSF and IFN-γ: p<0.0001) (Fig. 5A). GM-CSF appeared to have a minor effect on constitutive MCP-1 expression, with GM-CSF-treated macrophages producing approximately 2-fold more MCP-1 than untreated macrophages, but this difference was not significant (p = 0.6700) (Fig. 5A). Hence, untreated macrophages produced low MCP-1/low IL-8, macrophages treated with IFN-γ produced high MCP-1/low IL-8, macrophages treated with GM-CSF produced moderate MCP-1/high IL-8, and macrophages treated with IFN-γ and GM-CSF produced high MCP-1/high IL-8 (Fig. 5A).

After *P*. *aeruginosa* infection IFN-γ significantly boosted MCP-1 production, regardless of the presence of GM-CSF (IFN-γ: p = 0.0020; GM-CSF and IFN-γ: p = 0.0105) (Fig. 5A). In contrast, macrophages produced equivalent amounts of IL-8 after infection irrespective of cytokine treatment (Fig. 5A). Upon infection the median IL-8 expression by untreated macrophages and macrophages treated with IFN-γ was increased by 14-fold and 18-fold above baseline levels, respectively, while that by macrophages treated with GM-CSF in the absence and presence of IFN-γ only increased by 3 to 4-fold above baseline levels (Fig. 5B). These differences in fold increases are consistent with the levelling of IL-8 production among the different macrophage populations. In contrast, the fold increase in MCP-1 production upon *P*. *aeruginosa* infection did not differ among the differentially activated macrophages (Fig. 5B). IL-1β, IL-18, and MIP-1α expression in response to *P*. *aeruginosa* was not affected by macrophage activation (S9 Fig.).

**Fig 6:**
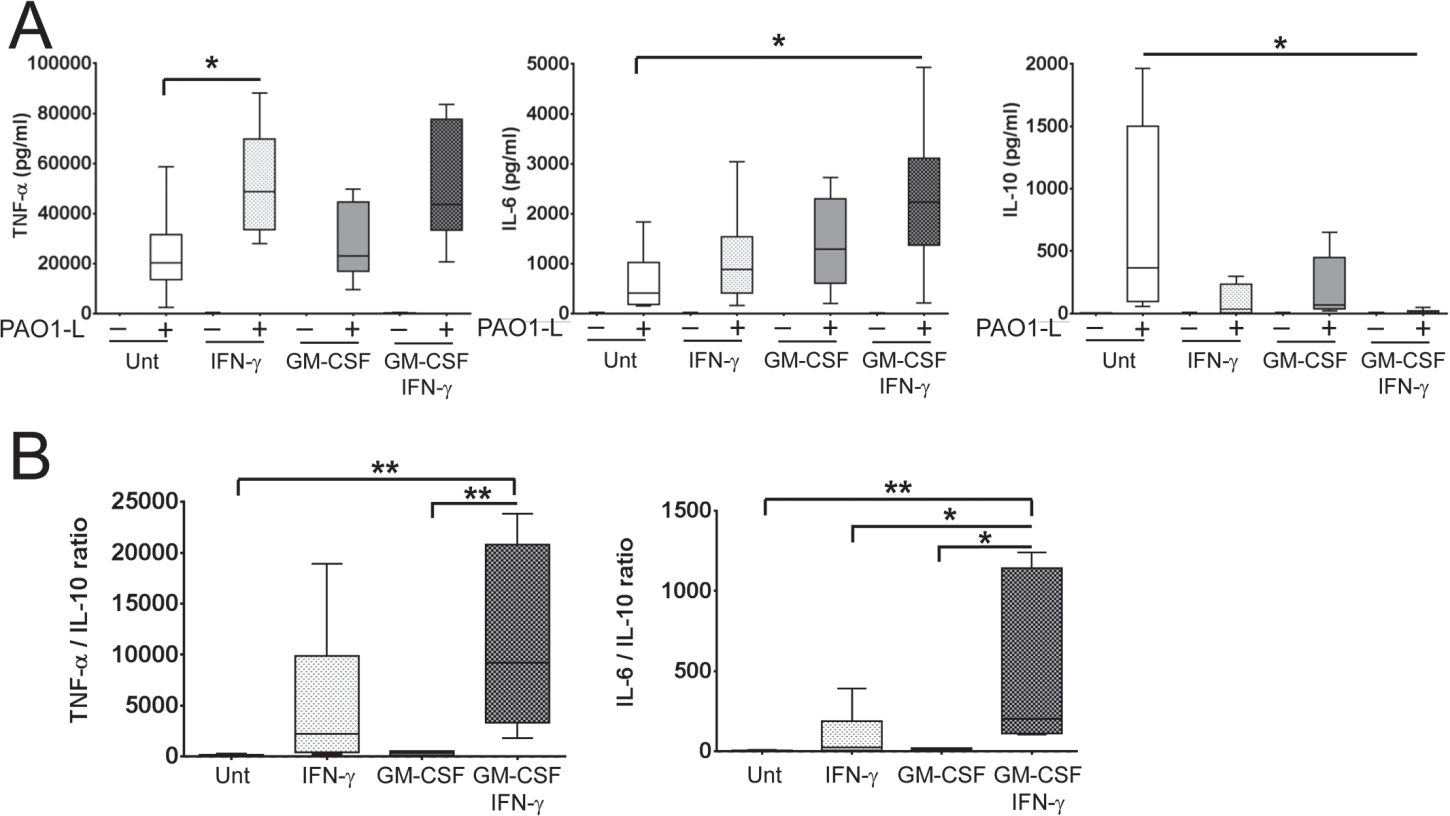
Activation with IFN-γ in the presence and absence of GM-CSF enhances the pro-inflammatory cytokine profile of macrophages in response to live *P*. *aeruginosa*. Macrophages were plated in X-Vivo 15, treated with IFN-γ in the presence and absence of GM-CSF for 48 h and infected with PAO1-L for 4 h. **A**. Macrophage activation upregulated the production of TNF-α and IL-6 and downregulated IL-10 production in response to PAO1-L infection. Negligible to no production of these cytokines was seen in uninfected macrophages under all activation conditions. **B**. Ratios of TNF-α vs. IL-10 and IL-6 vs IL-10 revealed that the combination of IFN-γ and GM-CSF produced the most inflammatory macrophage population. In donors where IFN-γ activation completely abrogated IL-10 expression, the minimum detectable limit of IL-10 for the assay (1.9 pg/ml) was used in order to calculate TNF-α/IL-10 and IL-6/IL-10 ratios. Graphs depict 5–95 percentile with median for n = 6 to 8. Significance calculated by one-way ANOVA with Tukey’s post test; * = p≤0.05, ** = p≤0.01. Unt: untreated macrophages; (-): uninfected; (+): infected.

Macrophage activation up-regulated TNF-α and IL-6 and down-regulated IL-10 production upon PAO1-L infection and differences between the effects of IFN-γ and GM-CSF were observed (Fig. 6A). TNF-α production was up-regulated approximately 2-fold by IFN-γ specifically; this was significant for macrophages treated with IFN-γ alone (p = 0.0378) (Fig. 6A). IL-6 production was weakly enhanced by IFN-γ or GM-CSF alone, but was significantly increased when used in combination (p = 0.0287) (Fig. 6A). Finally, while treatment with IFN-γ or GM-CSF alone did not reduce IL-10 production significantly (IFN-γ: ~11-fold, p = 0.0556; GM-CSF: ~5-fold, p = 0.2084), both cytokines in combination caused a significant 72-fold reduction (p = 0.0420) (Fig. 6A). In some donors, IL-10 was undetectable in infected cultures of macrophage treated with GM-CSF and IFN-γ. The effect of IFN-γ and GM-CSF on IL-6 expression appeared additive, while on IL-10 expression it appeared synergistic. TNF-α vs. IL-10 and IL-6 vs. IL-10 ratios revealed the following hierarchy regarding the inflammatory potential of activated macrophages in response to *P*. *aeruginosa* infection: untreated < treated with GM-CSF < treated with IFN-γ < treated with GM-CSF and IFN-γ (Fig. 6B). GM-CSF and IFN-γ did not affect *P*. *aeruginosa* growth in X-Vivo 15 based on results from independent growth curves and the quantification of cfu in bacteria-only control cultures done in parallel to macrophage infections (S10 Fig.).

These results illustrate the complementary capabilities of IFN-γ and GM-CSF for modulating the inflammatory potential of human macrophages under steady state conditions and in response to *P*. *aeruginosa* infection and support a role for macrophages in boosting inflammation in response to *P*. *aeruginosa* during an IFN-γ-dominated response, particularly in the presence of GM-CSF.

### IFN-γ in the presence and absence of GM-CSF does not alter *P. aeruginosa* growth or macrophage survival

**Fig 7:**
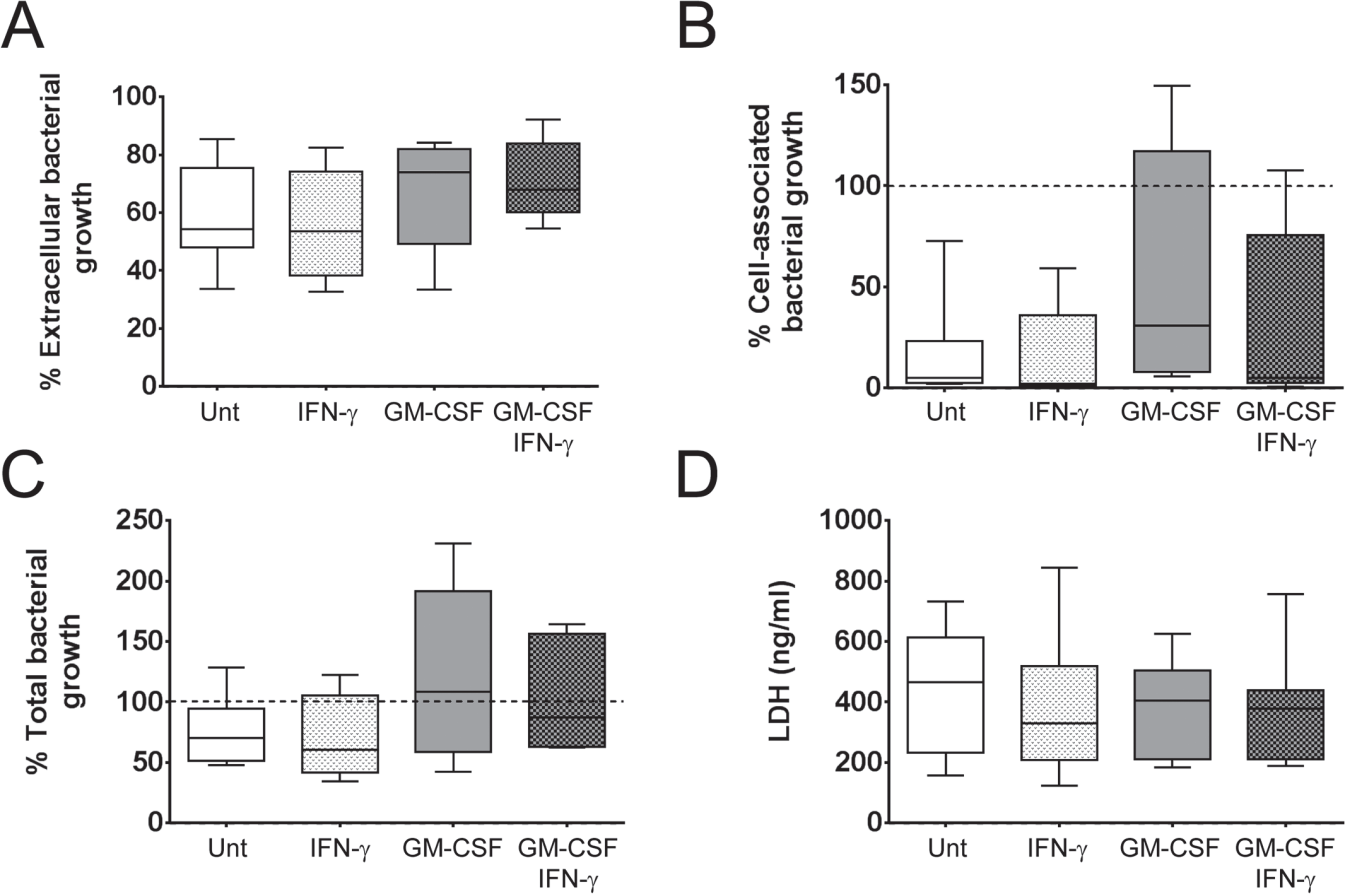
IFN-γ activation in the presence and absence of GM-CSF did not affect macrophage survival or *P*. *aeruginosa* growth. Macrophages were plated in X-Vivo 15, treated with IFN-γ in the presence and absence of GM-CSF for 48 h and infected with PAO1-L for 4 h. No significant differences were observed in the percentage of extracellular **(A)**, cell-associated **(B)**, and total **(C)** bacterial growth among cultures of differentially activated infected macrophages. The cell-associated fraction was found to be increased in certain donors. n = 6 to 10 for all bacterial growth data. **D**. Levels of LDH in infected macrophage supernatants were not significantly affected by macrophage activation, n = 6 to 10. Significance was calculated by one-way ANOVA with Tukey’s post test. Unt: untreated macrophages.

Treatment with IFN-γ in the presence and absence of GM-CSF did not affect the ability of human macrophages to restrict *P*. *aeruginosa* growth at 4 hpi. A consistent reduction in extracellular bacteria was observed in the presence of macrophages, irrespective of donor or macrophage treatment (Fig. 7A). As shown in Fig. 4A, in most instances the cell-associated bacterial fraction was minimal (Fig. 7B). However, in certain donors, extra growth of the cell-associated fraction was observed and compensated for the reduction in the extracellular fraction, such that in these cases the presence of macrophages did not affect the total amount of bacteria (Fig. 7C). Finally, comparison of LDH concentrations in supernatants from infected macrophages at 4 hpi revealed that activation of macrophages with IFN-γ in the presence and absence of GM-CSF did not significantly affect macrophage survival at this time point (IFN-γ: p = 0.9475; GM-CSF: p = 0.9438; GM-CSF and IFN-γ: p = 0.9001) (Fig. 7D). These results suggest that the altered cytokine / chemokines responses obtained upon activation with IFN-γ in the presence and absence of GM-CSF (Figs. 5 and 6) were not due to differences in cell survival or bacterial load and that cellular activation does not protect macrophages from *P*. *aeruginosa*-induced cytotoxicity nor affects *P*. *aeruginosa* growth in the presence of macrophages.

## Discussion

### Cytokine responses in CF PBMCs

This study corroborates the observation that systemic Th1 responses, based on levels of IFN-γ, are reduced in CF patients [5–7]. The reduced IFN-γ production in CF PBMCs appeared to be in response to general stimuli (PHA, SEB) rather than *P*. *aeruginosa*-specific (Fig. 1A). This might be as a consequence of a higher number of *P*. *aeruginosa*-specific circulating helper T cells in CF patients. In our study, both CF/Chronic PA and CF/Intermittent-Free PA PBMC cultures yielded reduced IFN-γ in response to PHA or SEB. Other studies differ on whether chronic *P*. *aeruginosa* infection correlates with IFN-γ responses. Brazova et al. reported similar IFN-γ production in LPS- or PHA-stimulated whole blood cultures from CF children irrespective of *P*. *aeruginosa* status [36]. However, Moser et al. observed that *P*. *aeruginosa* outer membrane protein (OMP)-stimulated PBMCs from *P*. *aeruginosa*-infected CF patients produced significantly less IFN-γ than those from *P*. *aeruginosa*-negative CF patients [5]. Hartl et al. found greater numbers of Th2 cells, higher levels of IL-4 and IL-13, and lower levels of IFN-γ in the bronchoalveolar lavage of CF patients chronically infected with *P*. *aeruginosa* compared to *P*. *aeruginosa*-free CF patients [7]. These discrepancies between studies may be due the use of different patient cohorts and classification criteria for *P*. *aeruginosa* infection status. For instance Moser et al. and Hartl et al. recruited patients who were free of *P*. *aeruginosa* infection rather than those intermittently infected or free of *P*. *aeruginosa* as in the study presented here. Robust Th1 responses to the mitogen PHA, correlated with better pulmonary status in CF patients, particularly in CF patients chronically infected with *P*. *aeruginosa* (Fig. 2). Moser et al. reported a similar significant correlation in their cohort of CF patients chronically infected with *P*. *aeruginosa* [5]. While reduced IFN-γ in CF samples is suggestive of a Th2 bias in CF PBMCs, we did not observe increased production of the Th2-associated cytokine IL-13 (S5 Fig.), nor did we detect any correlation between IFN-γ and IL-13 levels in response to PHA, SEB and PA Lo (data not shown). Our culture conditions did not support the detection of IL-4 but further work looking at IL-5 levels [18] could help to address this issue.

The reduced levels of IL-17A detected in CF cultures in response to all stimuli used (Fig. 1B) were surprising based on previous reports in support of IL-17-driven inflammation in CF [14–18]. It could be argued that IL-17A production at peripheral sites might not correlate with IL-17A produced by circulating immune cells upon stimulation; particularly because IL-17A-producing cells might be recruited to the lung or peripheral lymph nodes [14]. Bayes et al. detected *Pseudomonas*-specific Th1, Th17 and Th22 cells in both healthy and CF individuals and observed reduced percentages of Th17 memory CD4+ T cells specific for *P*. *aeruginosa* in the peripheral blood of CF patients compared to healthy controls [37]. Additionally IL-17A production in the lung could be mediated by a variety of cells. For instance, Tams et al. identified IL-17+ neutrophils, γδT cells and natural killer cells in addition to Th17 lymphocytes in bronchial biopsies of children with CF [17]. However our results are in clear disagreement with studies that identified increased numbers of circulating IL-17A-producing T cells in CF patients [16,18] and the intrinsic predisposition of CF T cells to differentiate into Th17 cells recently described [16].

The usefulness of IFN-γ and IL-17A production by PBMC as indicators of lung health in CF may be limited by factors such as the stimulus used. PHA-induced IFN-γ and IL-17A production in CF samples appears to be biologically relevant because of the positive and negative correlations between lung function and PHA-induced IFN-γ and IL17A production, respectively, observed for CF/Chronic PA samples (Fig. 2). In contrast IFN-γ and IL17A production in response to SEB or PA Lo did not show any correlation with lung function (data not shown), indicating a stimulus-dependent effect. While PHA induces polyclonal T cell activation in the presence of soluble factors, such as IL-6, produced by accessory cells [38] this would not be the case for SEB and *P*. *aeruginosa* lysate. The superantigen SEB is a toxin produced by *Staphylococcus aureus* [39] that cross-links the T cell receptor (TCR) on αβT cells bearing particular Vβ chains with non-cognate MHC molecules, thus allowing promiscuous T cell activation [39]. SEB can activate up to 20% of all αβ T cells (predominantly CD4+ T cells) therefore only a subpopulation of T cells was being probed in our assays. SEB is also capable of engaging iNKT cells through MHC class II, but not CD1d [40]. SEB requires expression of HLA-DR at the cell surface of monocytes and *P*. *aeruginosa* lysates require antigen processing and presentation by antigen presenting cells in the PBMC cultures. It is possible that CF monocytes could not effectively support SEB and/or *P*. *aeruginosa* lysates-mediated lymphocyte activation as they have been shown to express reduced levels of MHCII and might be impaired in antigen presentation [41].

Investigation of the behaviour of human PBMCs *in vitro* offers a useful non-invasive tool to assess the immune status of patients and its relationship with disease. In general, PBMC cultures are normalised for total viable cells which does not take into account potential differences in cellular composition. In this regard it is pertinent to refer to a recent work by Rieber et al. describing increased numbers of myeloid-derived suppressor cells (MDSC) in PBMCs of CF patients [42]. These potent immunosuppressive cells [43] were preferentially present in patients chronically infected with *P*. *aeruginosa*, in particular those infected with non-mucoid strains, and their percentage in total PBMCs from CF patients varied from less than 1% to 20%. Furthermore, the presence of MDSC correlated with improved lung function in CF patients infected with *P*. *aeruginosa*. CF-MDSC dampened production of several cytokines, chemokines and growth factors in stimulated PBMC cultures including IL-17A, IFN-γ, IL-13, IL-6, GM-CSF and IL-10 [42]. We propose that differences in patient characteristics, and in turn the cellular composition of the PBMC preparations such as T and B cell subsets [44,45], might account for some of the discrepancies observed, particularly in the case of IL-17A production. Two issues need to be taken into consideration: (i) the mean age of the cohort (6.1 in the case of Tiringer et al [18], 16 in the case of Rieber et al [42] vs. 27 in this study) as older patients, who would have been exposed to chronic inflammatory conditions for longer might have increased numbers of MDSC in circulation [46] and (ii) the type of *P*. *aeruginosa* isolates (e.g. flagellated vs. non-flagellated) as infection with flagellated *P*. *aeruginosa* was associated with MDSC induction in CF patients and flagellum induced the generation of MDSC [42].

### The potential contribution of human macrophages to maintenance of lung function during IFN-γ-dominated immune responses in the context of *P*. *aeruginosa* infection

In the study presented here it was next questioned how IFN-γ could positively impact on lung function in CF patients (Fig. 2). Preservation of organ function during infection results from a delicate balance between pro-inflammatory processes that promote clearance of infectious agents but have the potential to cause substantial tissue damage, and anti-inflammatory processes, aimed at minimising tissue damage and promote resolution that could compromise anti-microbial immunity. Thus IFN-γ could be beneficial in CF by promoting an inflammatory response capable of controlling infection while minimising lung damage. Macrophages play a central role in the regulation of inflammation and it was hypothesised that investigation of the effect of IFN-γ on the behaviour of macrophages during *P*. *aeruginosa* infection could provide information regarding the potential benefits of an IFN-γ-driven inflammatory response against *P*. *aeruginosa*. GM-CSF was included in the study because of its established activating effect on myeloid cell function [28,29] and the reported correlation between high serum GM-CSF with increased IFN-γ expression and better pulmonary function in CF patients [6]. Also, recent publications indicate that GM-CSF production by Th17 cells is responsible for their pathological role during chronic inflammation [47,48]. Although there are clear limitations when trying to translate *in vitro* findings to *in vivo* conditions it is possible to speculate regarding the biological consequences of the work presented here and make predictions that can be tested using more complex systems or clinical samples.

This manuscript shows that the response of macrophages to *P*. *aeruginosa* infection can be modulated by IFN-γ and GM-CSF and that both cytokines synergise to increase the inflammatory potential of macrophages in response to *P*. *aeruginosa*. In particular macrophages treated with IFN-γ produce more TNF-α and less IL-10 in response to *P*. *aeruginosa* infection than untreated macrophages and macrophages treated with GM-CSF. GM-CSF synergises with IFN-γ in reducing IL-10 synthesis by macrophages in response to infection. Hence it is possible that exposure of macrophages to IFN-γ and GM-CSF in the lung could trigger an aggressive inflammatory response characterised by high TNF-α/IL-10 and IL-6/IL-10 ratios (Fig. 6B) that promotes tissue damage. In agreement with this idea GM-CSF can be expressed in association with IFN-γ by human T cells during chronic inflammation [49]. The drastic reduction in IL-10 production induced by IFN-γ and GM-CSF could also be linked to the detrimental role of IL-17A in CF. GM-CSF can be produced by Th17 cells and if we consider IL-17A as a surrogate marker for the presence of GM-CSF, it could be speculated that the negative correlation between IL-17A levels and lung function in CF (Fig. 2) could be potentially caused by increased levels of GM-CSF in these patients. Further work would benefit from the identification of T cell subsets in the lungs of CF patients with and without *P*. *aeruginosa* infection with particular focus on T cells that co-express IFN-γ and GM-CSF and their relation to Th17 cells [49].

IFN-γ and GM-CSF can induce chemokine production in cultures of uninfected macrophages (Fig. 5); IFN-γ enhanced constitutive expression of the monocyte chemoattractant MCP-1 (Fig. 5), while GM-CSF upregulated the neutrophil chemoattractant IL-8. Each macrophage population under investigation appeared to produce a distinct chemokine pattern in the absence of infection: untreated macrophages low MCP-1/low IL-8, macrophages treated with IFN-γ high MCP-1/low IL-8, macrophages treated with GM-CSF moderate MCP-1/high IL-8, and macrophages treated with IFN-γ and GM-CSF high MCP-1/high IL-8. Hence, macrophages exposed to IFN-γ or GM-CSF could recruit monocytes and/or neutrophils, even before encountering a pathogen. In infected macrophages treatment with IFN-γ in the presence or absence of GM-CSF did not affect the production of IL-8 indicating that neutrophil recruitment might not be affected by these cytokines during infection. On the other hand, IFN-γ treatment could promote recruitment of monocytes by infected macrophages regardless of the presence of GM-CSF which might limit tissue destruction caused by neutrophils. The results from the present manuscript are in agreement with a previous report on the differential regulation of IL-8 and MCP-1 expression in human macrophages, with MCP-1 synthesis being induced in response to IFN-γ and IL-8 in response to LPS [50,51].

Activation with IFN-γ in the presence and absence of GM-CSF did not alter the production of the inflammasome-associated cytokines IL-1β or IL-18 (S9 Fig.). This is relevant in the context of *P*. *aeruginosa* respiratory infections as it has been demonstrated in murine acute *P*. *aeruginosa* pneumonia models that IL-1β and IL-18 released upon alveolar macrophage inflammasome activation are deleterious for the host [52–54]. Cellular responses to IFN-γ and GM-CSF are dominated by STAT-1 [55] and STAT-5 [56], respectively, with both cytokines conferring a ‘primed state’ to macrophages that enhances their ability to respond to stimulation. It would be of interest to characterise the interplay between the signalling pathways triggered by IFN-γ and GM-CSF in order to determine the molecular basis for their additive and synergistic effects with particular emphasis on how they cooperate to regulate IL-10 expression.

Macrophages could potentially control the growth of *P*. *aeruginosa* through phagocytosis, production of reactive oxygen species, release of hydrolases and iron sequestration [57,58]. Macrophage activation with IFN-γ in the presence and absence of GM-CSF did not affect bacterial growth or protect macrophages from *P*. *aeruginosa*-induced cytotoxicity (Figs. 4B-C and 7D). Hence, an IFN-γ-dominated (Th1) immune response is unlikely to safeguard lung function by promoting the ability of macrophages to control *P*. *aeruginosa* infection or by increasing resistance of macrophages to the cytotoxic effect of *P*. *aeruginosa*. This is in agreement with a previous study describing a negative effect of IFN-γ on the phagocytosis and killing of *P*. *aeruginosa* by human macrophages [59]. The inability of macrophages treated with GM-CSF to restrict *P*. *aeruginosa* growth contrasts with the role played by this cytokine in protection against lethal *P*. *aeruginosa* pneumonia in vaccinated neutropenic mice [60]. It is possible that the protective role of GM-CSF in the murine pneumonia model could be facilitated by the presence of opsonins such as antibodies specific for *P*. *aeruginosa* or lung collectins, such as SP-A, which could promote phagocytic uptake and cellular activation, or the accumulation of monocytes/macrophages in the infected lungs of vaccinated animals [60].

There was also the possibility of *P*. *aeruginosa* changing to a senescent growth programme in the presence of macrophages as radical oxygen species and anti-microbial peptides have been shown to promote mucoidy in *P*. *aeruginosa in vitro* [61]. To address this, planktonic and cell-associated bacteria were quantified independently after infection. Microscopic analysis of infected cultures showed stepwise destruction of the macrophage monolayer with bacterial microcolonies observed within areas of cellular damage surrounded by healthy cells (S8 Fig.). This suggests that macrophage death was mediated by direct bacterial contact [62] and that secreted virulence factors might have not reached cytotoxic concentrations. Macrophage death in most cases correlated with the total bacterial burden (data not shown). The high expression of IL-1β and IL-18 (Fig. 3C and S9 Fig.) and nuclear condensation observed at 6 hpi (Fig. 4C) suggest that macrophages died by pyroptosis (inflammatory cell death caused by caspase-1 activation [63]). This would agree with other reports of *P*. *aeruginosa* type 3 secretion system inducing pyroptosis in macrophages via activation of the NLRC4 inflammasome [62]. Microcolony formation in the current study indicates transition from planktonic to sessile growth and might account for the increased cell-associated growth of *P*. *aeruginosa* in several experiments (Fig. 7B). Preliminary evidence suggests this may be donor-dependent. We propose that these changes in bacterial behaviour could be caused by the inflammatory conditions prevalent in these particular infections and be related to the levels of reactive oxygen species or anti-microbial peptides present [61].

In conclusion, we have observed that IFN-γ production by CF PBMCs positively correlated with lung function, particularly in patients chronically infected with *P*. *aeruginosa*, while IL-17A levels tended to correlate negatively with lung function, with this trend becoming significant in patients chronically infected with *P*. *aeruginosa*. These results are in agreement with IFN-γ and IL-17A playing protective and detrimental roles, respectively, in CF. In addition, our study provides information regarding parameters that need to be taken into consideration when looking at systemic immune responses in clinical samples. For instance, our results illustrate the importance of the stimuli used for activation of immune cells. Stimuli that require support from antigen presenting cells for T cell activation in the form of cytokines and/or high surface MHCII expression would provide a more comprehensive evaluation of the immunological status of patients as T cell responses are highly dependent on the activation state of antigen presenting cells. This would be lost when employing anti-CD3 and CD28 antibodies to stimulate T cells which would induce polyclonal activation of all T cells but will only allow the analysis of the potential levels of T cell-derived cytokines that can be produced in patients.

Results using human macrophages infected with *P*. *aeruginosa* indicate that IFN-γ might promote an inflammatory response characterised by a balanced production of pro- and anti-inflammatory cytokines and recruitment of mononuclear cells. This is agreement with early work showing the ability of IFN-γ to decrease the inflammatory response in chronic *P*. *aeruginosa* pneumonia in rats [8]. In contrast IFN-γ in combination with GM-CSF might promote tissue damage because of a reduced anti-inflammatory response, unable to safeguard tissues from the deleterious effects of inflammation.

Future work in this area will benefit from the use of isolated tissue macrophages from healthy donors and CF patients and clinical isolates of *P*. *aeruginosa*. It would also be pertinent to investigate the effect of macrophage-derived cytokines and other inflammatory mediators on other immune cells such as neutrophils. Particular emphasis should be placed in establishing activation regimes consisting of specific combinations of effector cytokines that promote the microbicidal capabilities of immune cells but minimise the potential for tissue damage. Co-cultures of phagocytes (macrophages and neutrophils) exposed to different combination of cytokines would provide a highly valuable tool to perform preclinical studies towards this aim.

## Supporting Information

S1 Fig Characterisation of human monocyte-derived macrophages differentiated in suspension (Teflon bottles) for 7 days. A. CD68 and mannose receptor (MR) expression in macrophages demonstrated by immunofluorescence. B. Flow cytometric analysis showed that CD14, CD16, HLA-DR, and MR surface expression was significantly upregulated upon monocyte (open circles) differentiation into macrophages (open squares). MFI = mean fluorescence intensity. Significance was calculated by unpaired Student’s t test for CD14, HLA-DR, and mannose receptor, and Mann-Whitney test for CD16. C. Reactive oxygen species production by zymosan-stimulated macrophages differentiated in M-CSF or GM-CSF.
(TIF)

S2 Fig PAO1-L growth in LB broth and X-Vivo 15 is similar under oxygenated conditions. PAO1-L was cultured in LB broth or X-Vivo 15 at 37°C, 200 rpm and OD_600nm_ of the cultures measured at the times indicated.
(TIF)

S3 Fig PAO1-N expresses quorum sensing molecules and virulence factors in X-Vivo 15 but the las and rhl systems are lower than in LB. PAO1-N (Nottingham subline) reporter strains carrying lux promoter fusions (see S1 Table) were used to determine the expression of quorum sensing and virulence factor genes in LB broth and X-Vivo 15. Luminescence and OD_600nm_ were measured for each culture every 30 min. To negate any differences in luminescence due to differences in growth of the reporter strains, gene expression was reported as luminescence (relative light units, RLU) divided by OD_600nm_ for that culture. Only every second data point is plotted for ease of viewing. Data presented are mean of three independent experiments. Bacterial strains are described in S1 Table.
(TIF)

S4 Fig LDH released by PAO1-L upon freezing and thawing and sonication to lyse cells. Three independently prepared mid-log phase cultures of PAO1-L in X-Vivo 15 at a density of 5 x 10^8^ cfu/ml (which was much higher than the total bacterial load ever achieved in any of the in vitro infection assays used in this study) released on average only 12.2 ± 3.6 ng/ml LDH (mean ± SD, n = 3) upon freezing and thawing followed by sonication.
(TIF)

S5 Fig IL-13 (A) and IL-10 (B) production by healthy and CF PBMCs stimulated with PHA, SEB or *P*. *aeruginosa* lysates. Graphs depict 5–95 percentile with median. For IL-10 (all stimuli), Healthy controls: n = 13, CF / Intermittent-Free PA and CF / Chronic PA: n = 15. For IL-13 (SEB, PA Hi, PA Lo), Healthy controls: n = 13, CF / Intermittent-Free PA: n = 15, CF / Chronic PA: n = 11, while for IL-13 (PHA): Healthy controls: n = 7, CF / Intermittent-Free PA: n = 13, CF / Chronic PA: n = 9 as IL-13 production by the remaining donors under this condition was above the standard range of the assay. Significance calculated by Kruskal-Wallis test with Dunn’s post test. * = p≤0.05, ** = p≤0.01.
(TIF)

S6 Fig Lung function in CF patients. CF / Intermittent-Free PA patients demonstrated significantly better lung function than CF / Chronic PA patients. This was particularly evident when pulmonary function was described as a percentage of predicted FEV1. Data presented are mean ± SD. Significance calculated by an unpaired Student’s t test.
(TIF)

S7 Fig Correlation between lung function and IFN-γ and IL-17A production in response to PHA in CF / Intermittent-Free PA patients. Correlation between IFN-γ and IL-17A production in response to PHA and lung function was non-significant in the subset of intermittent-free CF patients. Correlation calculated by Spearman rank test.
(TIF)

S8 Fig Analysis of PAO1-L-infected macrophage cultures by light microscopy. PAO1-L clusters can be observed at 2 hpi (black arrows) and increase in number and size at later times post-infection. Magnification = 400x.
(TIF)

S9 Fig Activation of human macrophages with IFN-γ in the presence and absence of GM-CSF does not significantly alter the expression of IL-1β, IL-18, and MIP-1α in response to PAO1-L at 4 hpi. Significance was calculated by one-way ANOVA with Tukey’s post test. IL-1β: p = 0.8320, n = 6 for +/- IFN-γ and n = 4 for GM-CSF +/- IFN-γ. IL-18: p = 0.9582, n = 6 for IFN-γ, n = 4 for GM-CSF +/- IFN-γ. MIP-1α: p = 0.3786, n = 6 for +/- IFN-γ and n = 4 for GM-CSF +/- IFN-γ. Unt: untreated controls.
(TIF)

S10 Fig PAO1-L growth in X-Vivo 15 under stationary conditions is not affected by the addition of macrophage-activating cytokines. A. Bacteria were cultured in a 96-well microtitre plate at 37°C for 24 h in a Tecan Infinite M1000 PRO plate reader which measured the absorbance (OD_600nm_) of the cultures every 30 min. Only every second data point is plotted for ease of viewing. Data presented are mean ± SD for two experiments. B. Number of cfu in bacteria only wells at 4 hpi during macrophage infection assays. No significant difference was observed in the presence of different cytokines (p = 0.4890, one-way ANOVA with Tukey’s post test).
(TIF)

S1 Table *P*. *aeruginosa* strains used in this study. (TIF)
