# Supplementary figures and images for: Granulocyte-Macrophage Colony Stimulatory Factor Enhances the Pro-Inflammatory Response of Interferon-γ-Treated Macrophages to Pseudomonas aeruginosa Infection
Source: PLoS One. 2015 Feb 23;10(2):e0117447. doi: 10.1371/journal.pone.0117447 (PMC4338139; doi:10.1371/journal.pone.0117447)

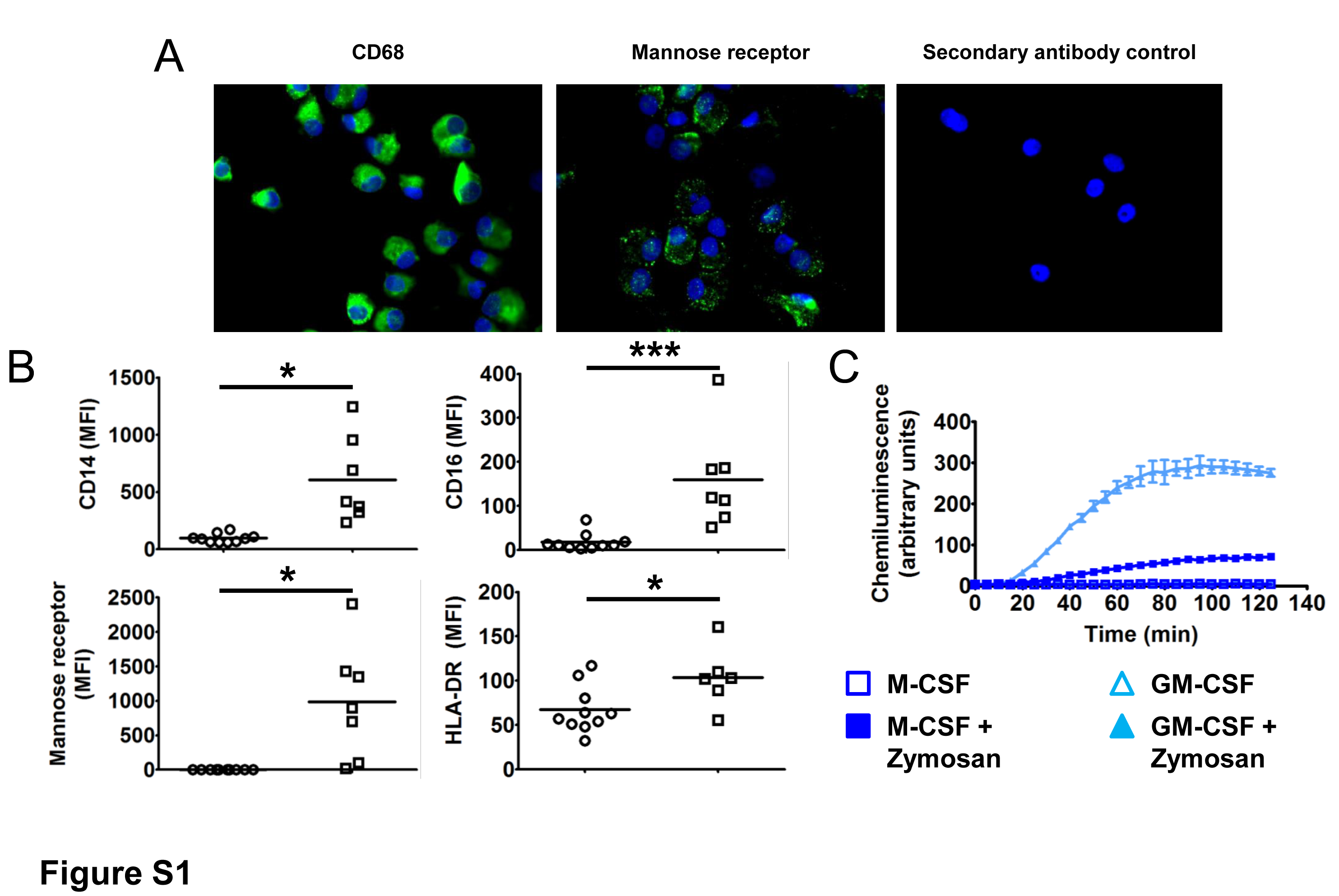

Supplement: S1 Fig — A. CD68 and mannose receptor (MR) expression in macrophages demonstrated by immunofluorescence. B. Flow cytometric analysis showed that CD14, CD16, HLA-DR, and MR surface expression was significantly upregulated upon monocyte (open circles) differentiation into macrophages (open squares). MFI = mean fluorescence intensity. Significance was calculated by unpaired Student’s t test for CD14, HLA-DR, and mannose receptor, and Mann-Whitney test for CD16. C. Reactive oxygen species production by zymosan-stimulated macrophages differentiated in M-CSF or GM-CSF. (TIF) [file pone.0117447.s001.tif]

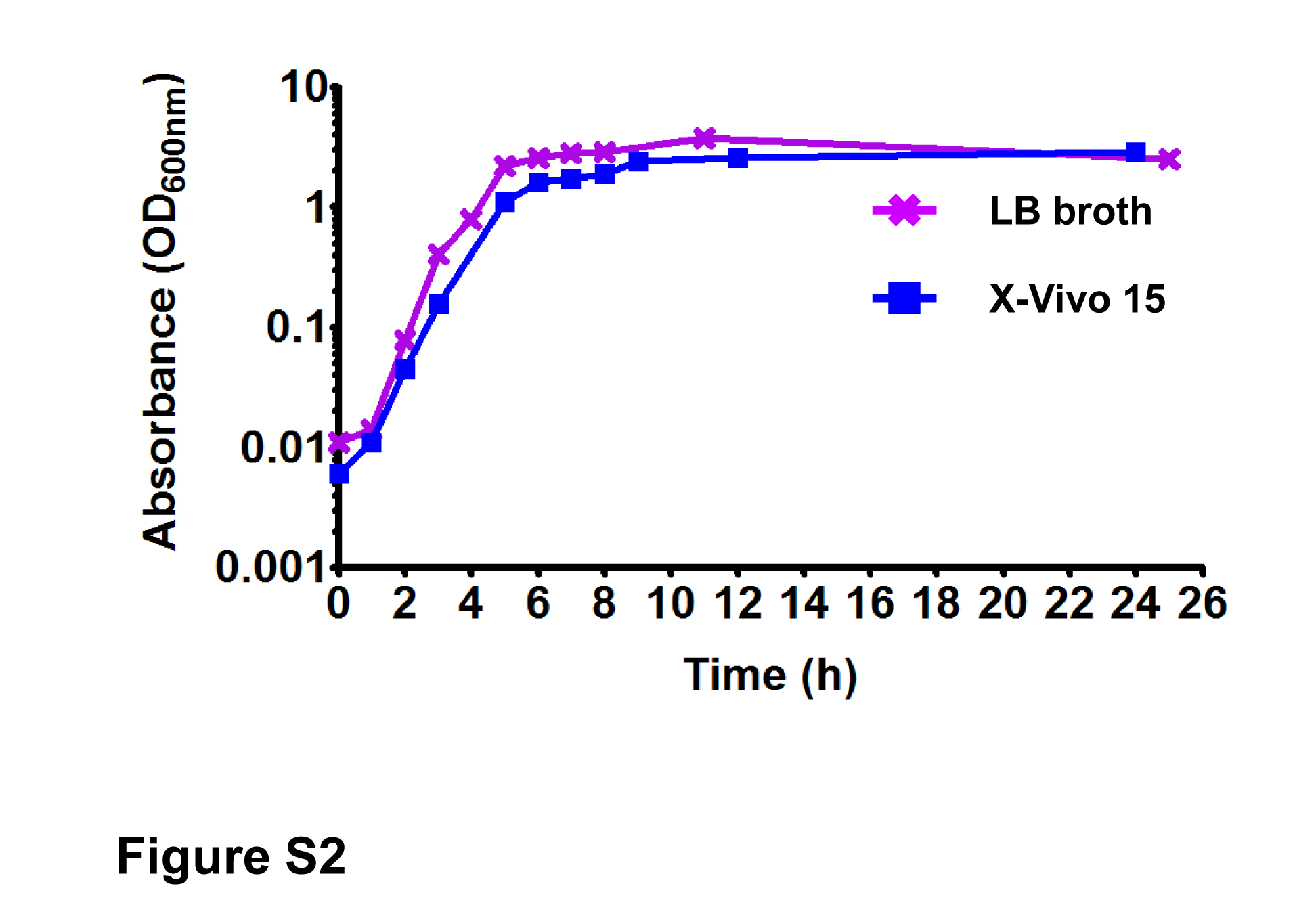

Supplement: S2 Fig — PAO1-L was cultured in LB broth or X-Vivo 15 at 37°C, 200 rpm and OD600nm of the cultures measured at the times indicated. (TIF) [file pone.0117447.s002.tif]

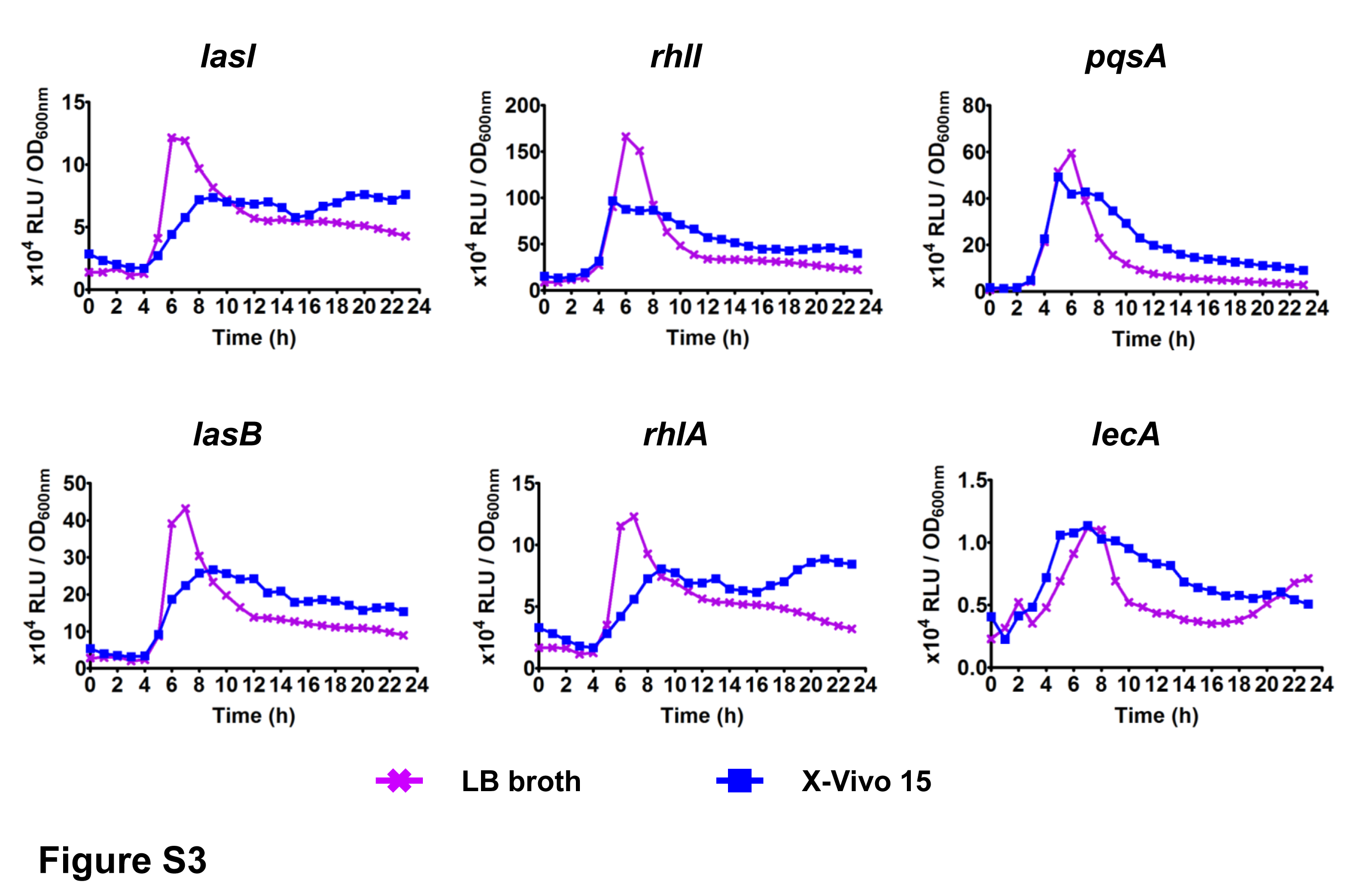

Supplement: S3 Fig — PAO1-N (Nottingham subline) reporter strains carrying lux promoter fusions (see S1 Table) were used to determine the expression of quorum sensing and virulence factor genes in LB broth and X-Vivo 15. Luminescence and OD600nm were measured for each culture every 30 min. To negate any differences in luminescence due to differences in growth of the reporter strains, gene expression was reported as luminescence (relative light units, RLU) divided by OD600nm for that culture. Only every second data point is plotted for ease of viewing. Data presented are mean of three independent experiments. Bacterial strains are described in S1 Table. (TIF) [file pone.0117447.s003.tif]

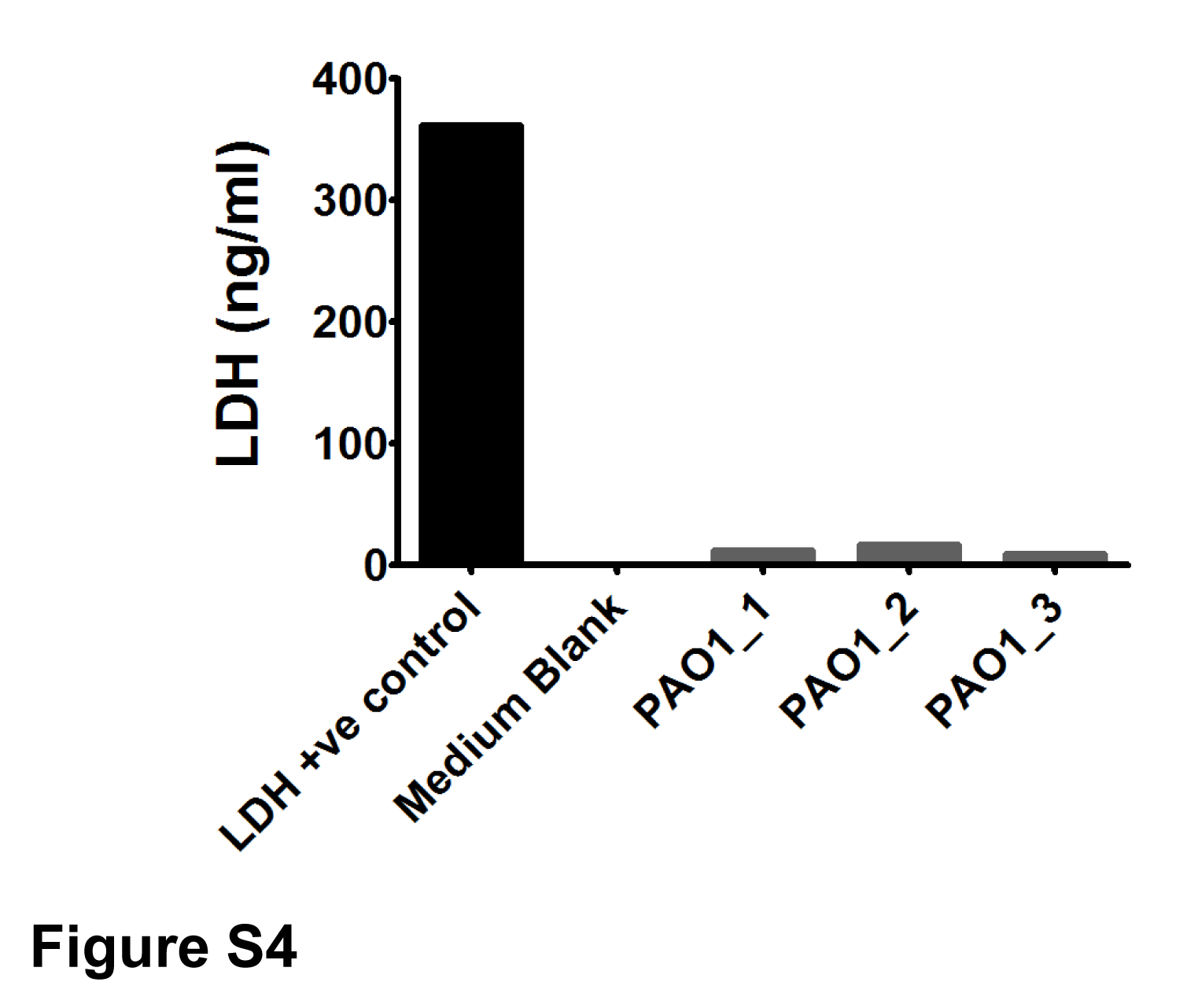

Supplement: S4 Fig — Three independently prepared mid-log phase cultures of PAO1-L in X-Vivo 15 at a density of 5 x 10^8^ cfu/ml (which was much higher than the total bacterial load ever achieved in any of the in vitro infection assays used in this study) released on average only 12.2 ± 3.6 ng/ml LDH (mean ± SD, n = 3) upon freezing and thawing followed by sonication. (TIF) [file pone.0117447.s004.tif]

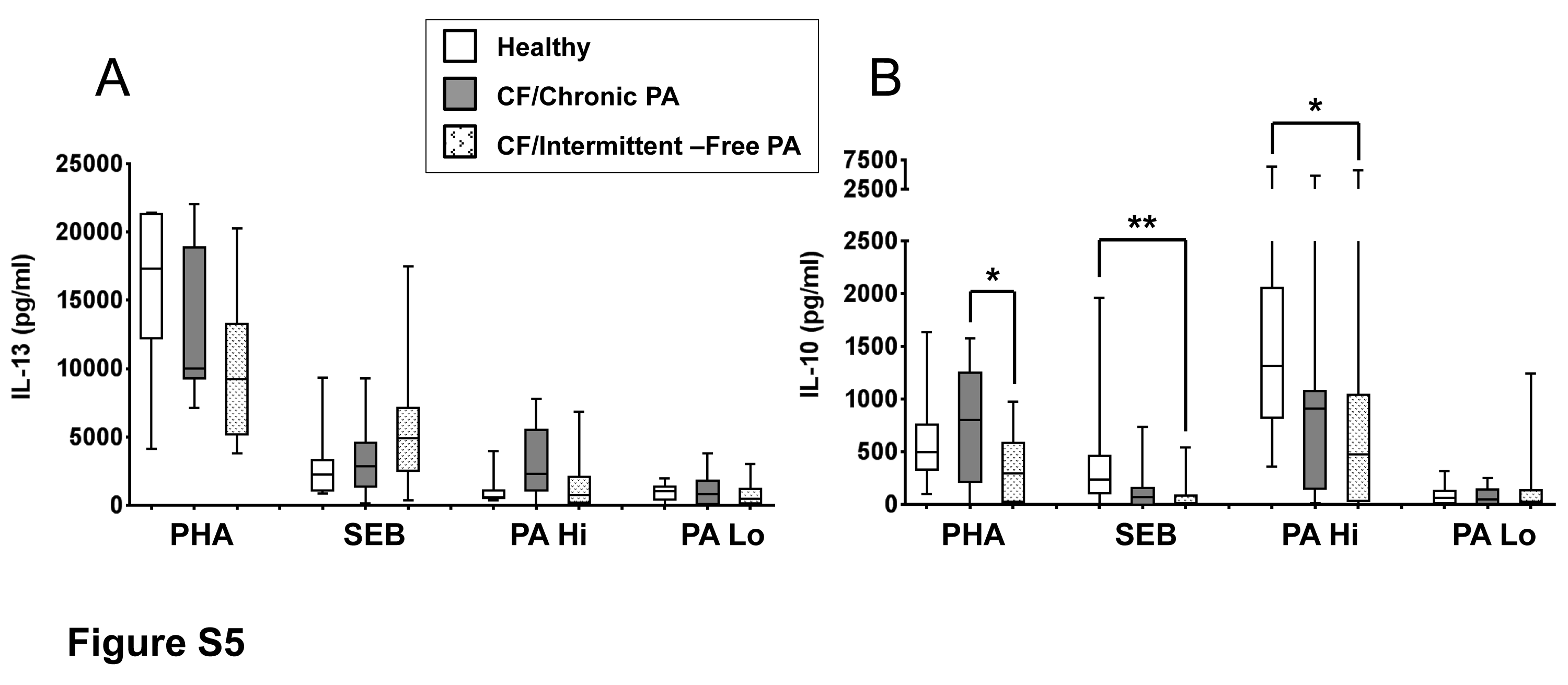

Supplement: S5 Fig — Graphs depict 5–95 percentile with median. For IL-10 (all stimuli), Healthy controls: n = 13, CF / Intermittent-Free PA and CF / Chronic PA: n = 15. For IL-13 (SEB, PA Hi, PA Lo), Healthy controls: n = 13, CF / Intermittent-Free PA: n = 15, CF / Chronic PA: n = 11, while for IL-13 (PHA): Healthy controls: n = 7, CF / Intermittent-Free PA: n = 13, CF / Chronic PA: n = 9 as IL-13 production by the remaining donors under this condition was above the standard range of the assay. Significance calculated by Kruskal-Wallis test with Dunn’s post test. * = p≤0.05, ** = p≤0.01. (TIF) [file pone.0117447.s005.tif]

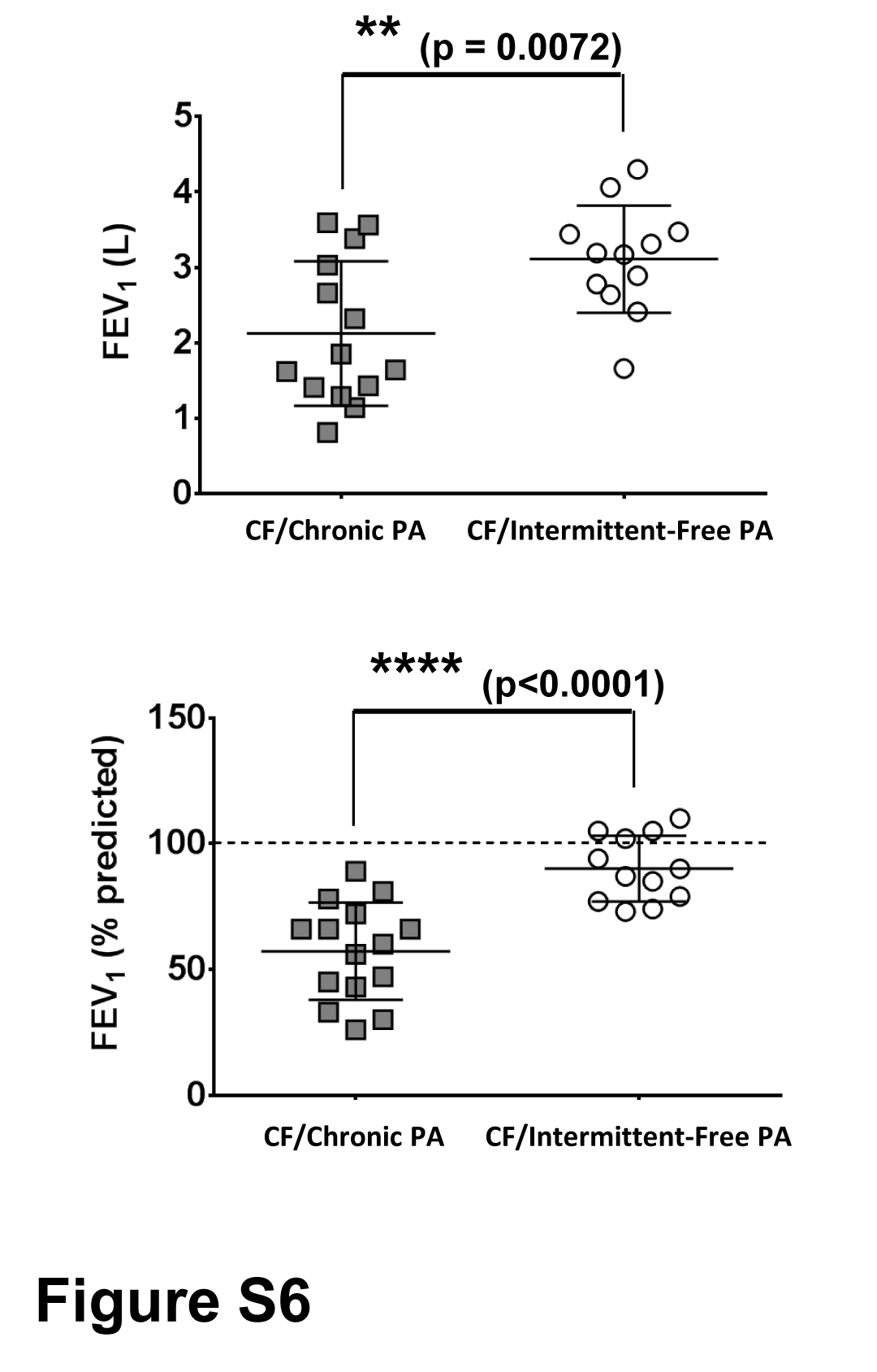

Supplement: S6 Fig — CF / Intermittent-Free PA patients demonstrated significantly better lung function than CF / Chronic PA patients. This was particularly evident when pulmonary function was described as a percentage of predicted FEV1. Data presented are mean ± SD. Significance calculated by an unpaired Student’s t test. (TIF) [file pone.0117447.s006.tif]

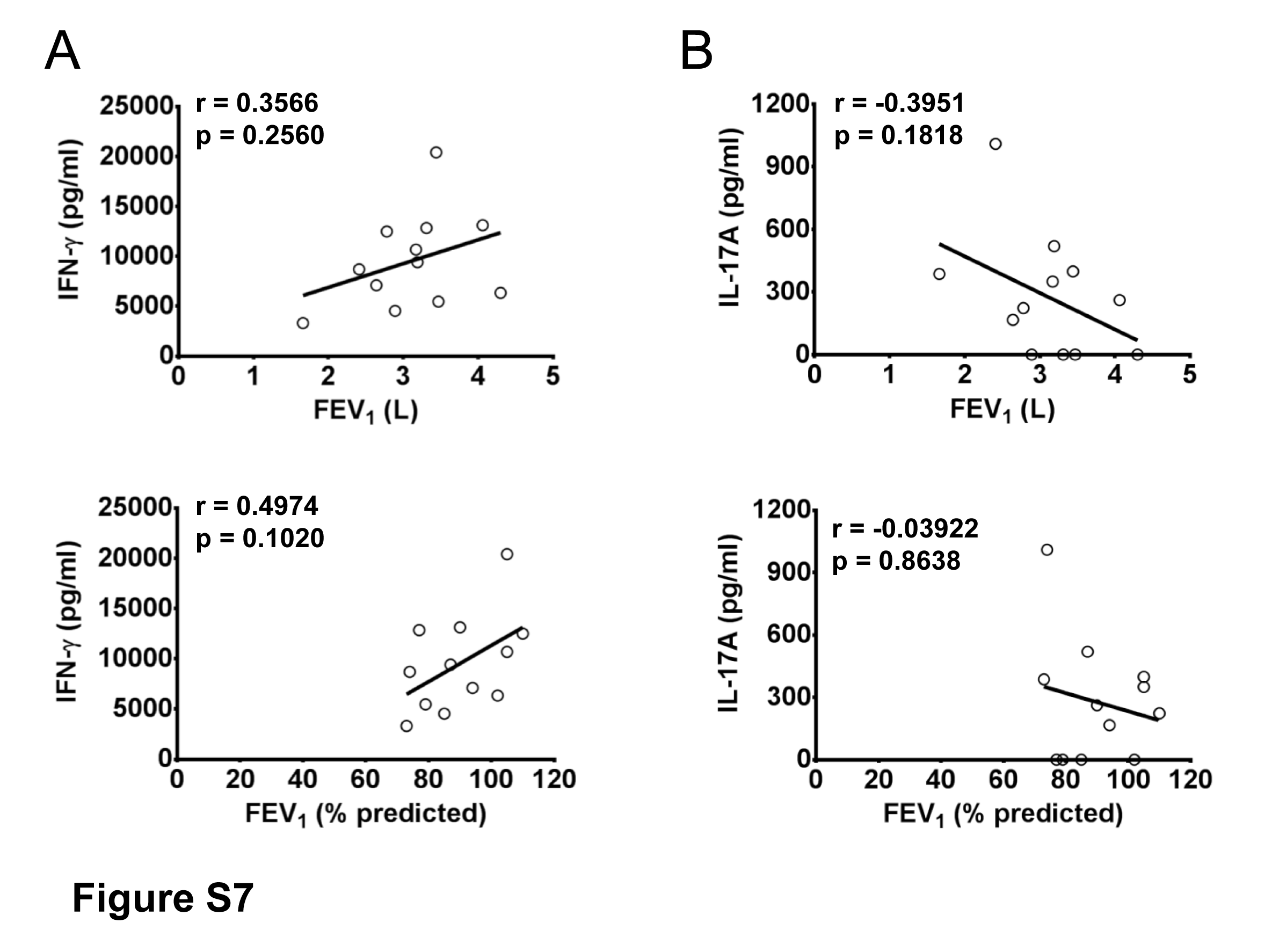

Supplement: S7 Fig — Correlation between IFN-γ and IL-17A production in response to PHA and lung function was non-significant in the subset of intermittent-free CF patients. Correlation calculated by Spearman rank test. (TIF) [file pone.0117447.s007.tif]

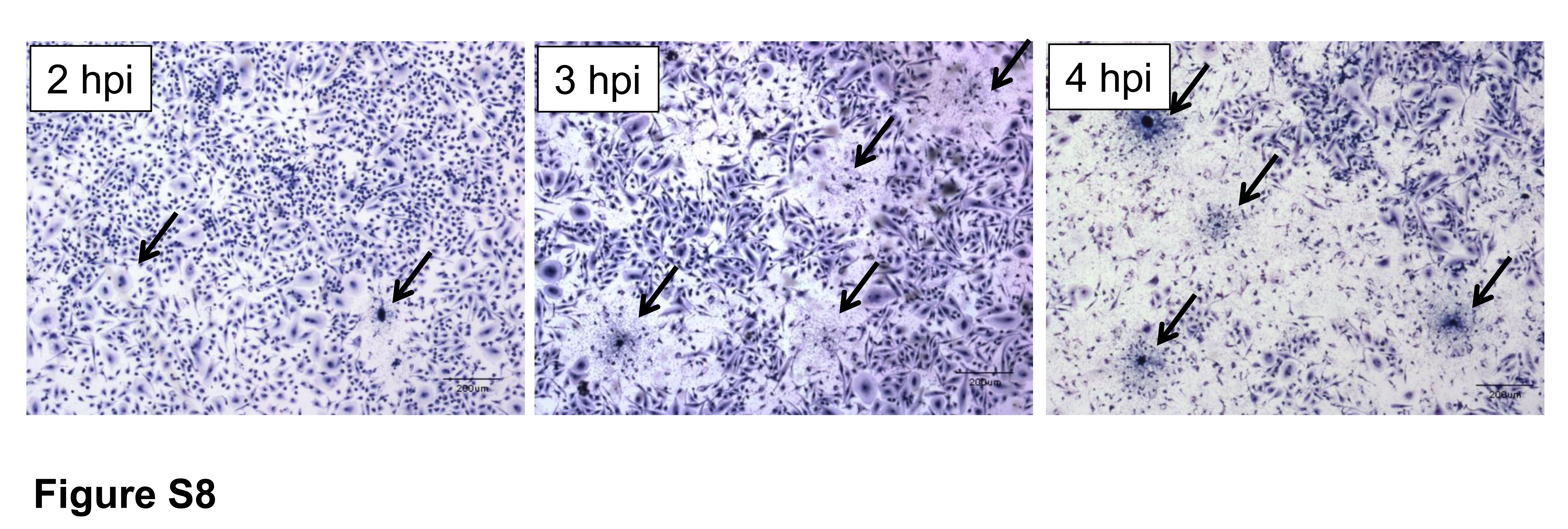

Supplement: S8 Fig — PAO1-L clusters can be observed at 2 hpi (black arrows) and increase in number and size at later times post-infection. Magnification = 400x. (TIF) [file pone.0117447.s008.tif]

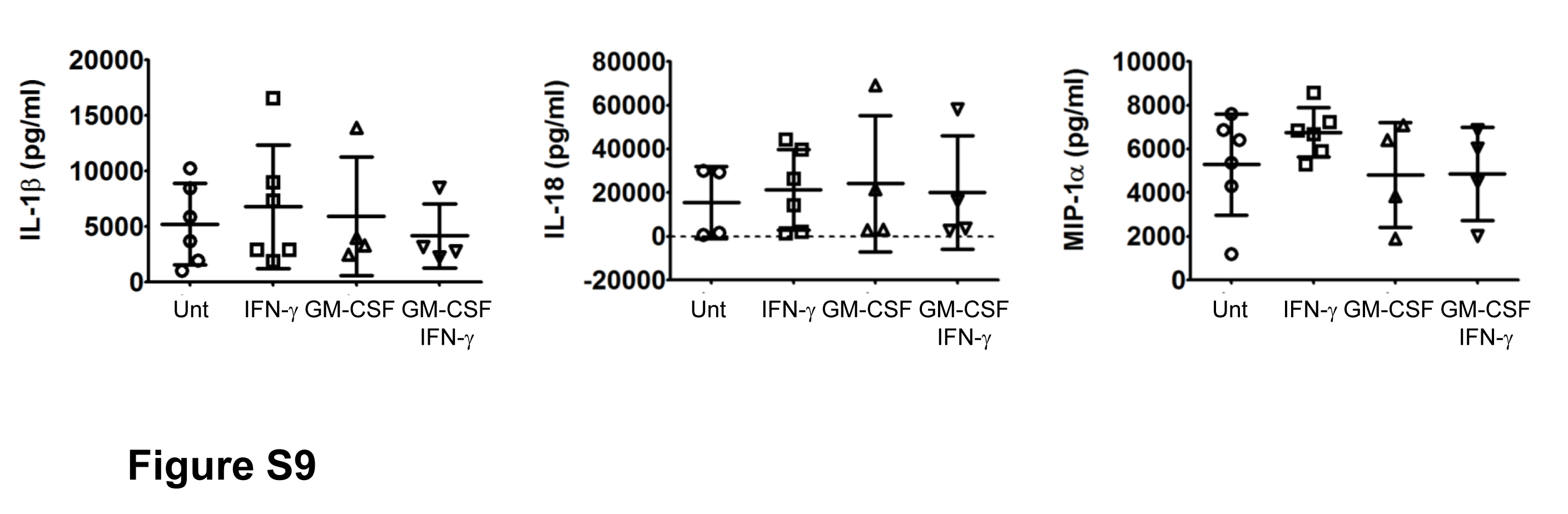

Supplement: S9 Fig — Significance was calculated by one-way ANOVA with Tukey’s post test. IL-1β: p = 0.8320, n = 6 for +/- IFN-γ and n = 4 for GM-CSF +/- IFN-γ. IL-18: p = 0.9582, n = 6 for IFN-γ, n = 4 for GM-CSF +/- IFN-γ. MIP-1α: p = 0.3786, n = 6 for +/- IFN-γ and n = 4 for GM-CSF +/- IFN-γ. Unt: untreated controls. (TIF) [file pone.0117447.s009.tif]

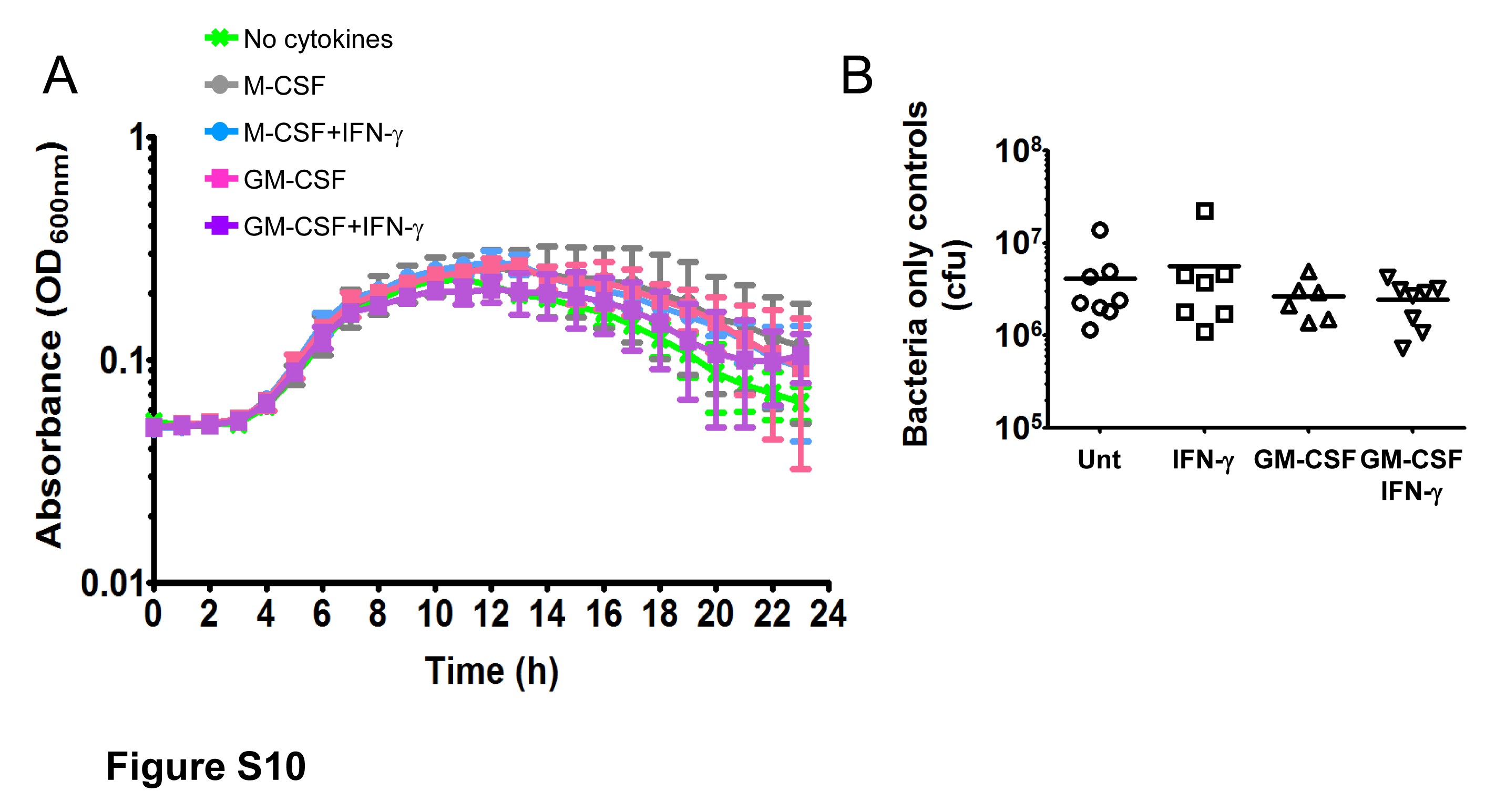

Supplement: S10 Fig — A. Bacteria were cultured in a 96-well microtitre plate at 37°C for 24 h in a Tecan Infinite M1000 PRO plate reader which measured the absorbance (OD600nm) of the cultures every 30 min. Only every second data point is plotted for ease of viewing. Data presented are mean ± SD for two experiments. B. Number of cfu in bacteria only wells at 4 hpi during macrophage infection assays. No significant difference was observed in the presence of different cytokines (p = 0.4890, one-way ANOVA with Tukey’s post test). (TIF) [file pone.0117447.s010.tif]

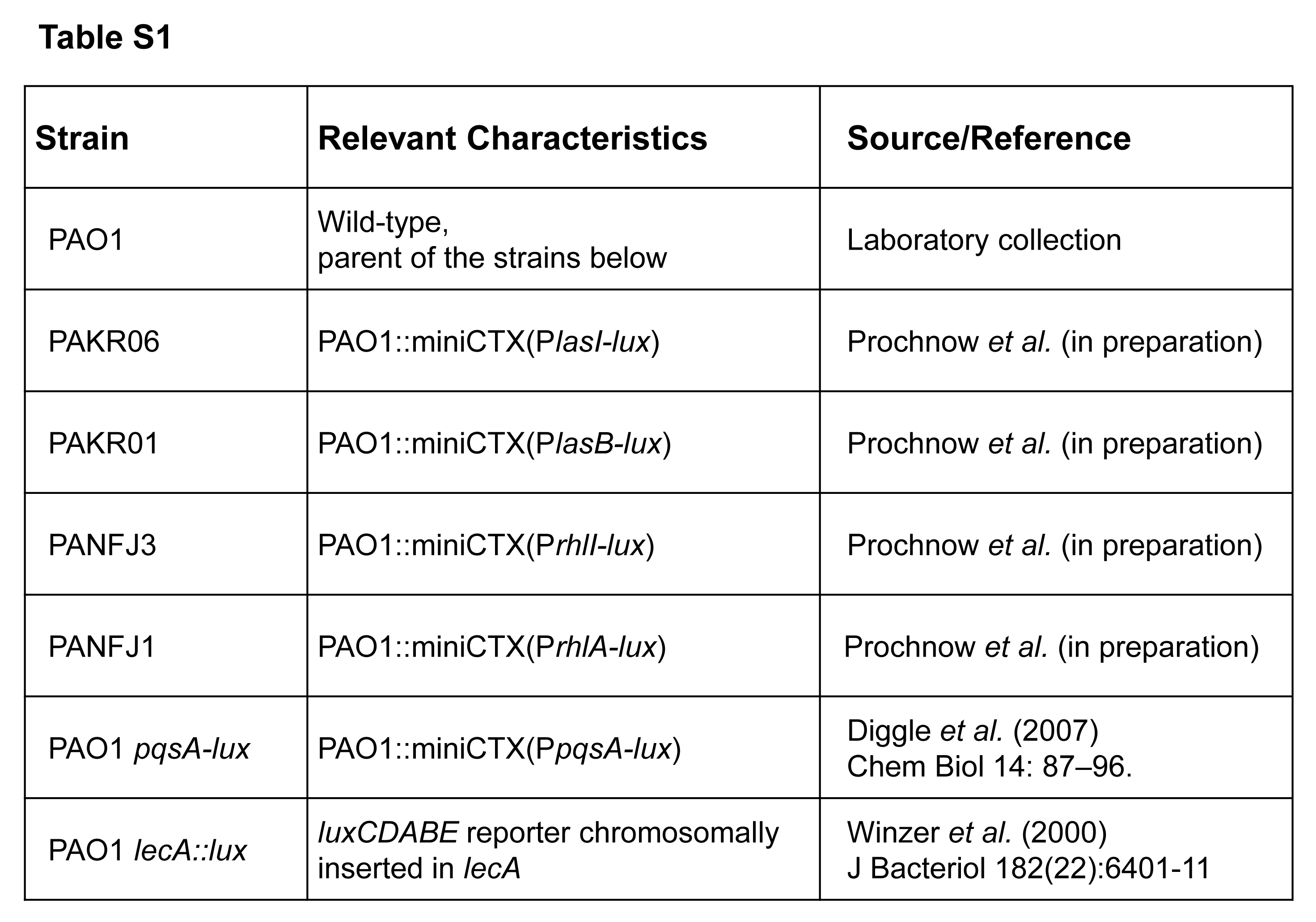

Supplement: S1 Table — (TIF) [file pone.0117447.s011.tif]
